# Synthesis, Polymorphism and Thermal Decomposition Process of (n-C_4_H_9_)_4_N*RE*(BH_4_)_4_ for *RE* = Ho, Tm and Yb

**DOI:** 10.3390/ma14061329

**Published:** 2021-03-10

**Authors:** Wojciech Wegner, Tomasz Jaroń

**Affiliations:** 1 College of Inter-Faculty Individual Studies in Mathematics and Natural Sciences, University of Warsaw, Banacha 2c, 02-097 Warsaw, Poland; 2 Centre of New Technologies, University of Warsaw, Banacha 2c, 02-097 Warsaw, Poland

**Keywords:** borohydrides, lanthanides, hydrogen storage, crystal structures, polymorphism, thermal stability, rare-earth elements

## Abstract

In total, three novel organic derivatives of lanthanide borohydrides, n-But_4_N*RE*(BH_4_)_4_ (TBA*RE*B), *RE* = Ho, Tm, Yb, have been prepared utilizing mechanochemical synthesis and purified via solvent extraction. Studies by single crystal and powder X-ray diffraction (SC-XRD and PXRD) revealed that they crystalize in two polymorphic forms, α- and β-TBA*RE*B, adopting monoclinic (*P*2_1_/c) and orthorhombic (*P*nna) unit cells, previously found in TBAYB and TBAScB, respectively. Thermal decomposition of these compounds has been investigated using thermogravimetric analysis and differential scanning calorimetry (TGA/DSC) measurements, along with the analysis of the gaseous products with mass spectrometry (MS) and with analysis of the solid decomposition products with PXRD. TBAHoB and TBAYbB melt around 75 °C, which renders them new ionic liquids with relatively low melting points among borohydrides.

## 1. Introduction

Metal borohydrides constitute a continuously growing family of chemical compounds. Although the dominant applicability of this group of compounds remains linked with their reducing properties (exploited mostly for NaBH_4_ and for LiBH_4_ [1]), the recent expansion of their chemistry is related to other potential applications. In the last two decades, due to extremely high hydrogen content of a BH_4_^-^ anion (>27 wt.%), such interest was predominantly stimulated by their prospective use as chemical hydrogen storage materials for supply of the fuel cells installed in vehicles [2,3,4,5,6,7,8,9]. Although most of studies focus on examining of the process of hydrogen release, several reports indicate at least some degree of reversibility of such systems, which is crucial for their application, e.g., [10]. Various borohydrides can also serve as precursors of metal borides [11,12,13,14,15] or boron nitride [16], and some of them have been explored as prospective solid-state electrolytes in Li^+^ batteries [17,18,19,20,21] or luminescent and magnetic materials [22,23], while the borohydride complexes of rare-earth elements were also tested for organic catalysis [24,25].

Several methods have been proposed to modify the physico-chemical properties of borohydride-based materials, among which substitution of the cations and formation of mixed-cation or mixed-anion compounds appeared most common [26,27,28]. Consequently, novel synthetic approaches have been developed to broaden the scope of available borohydride-based systems showing potentially interesting properties [22,29,30,31,32,33,34]. Among the methods available for preparation of the mixed-cation borohydrides in a solvent-free form either those based on mechanochemically-driven addition or metathesis, or on a solvent-mediated metathesis reaction reveal general applicability to diverse systems. While purification of the products of the mechanochemical synthesis may often be problematic, the approach utilizing weakly coordinating anions [35] and the solvents of low basicity [36] leads to mixed-cation borohydrides of rather high purity [31,32,37], e.g.,

[*Cat*]Y(BH_4_)_4_ + Cs[*An*] → CsY(BH_4_)_4_↓ + [*Cat*][*An*] (1)

The symbols [*Cat*] and [*An*] in Equation (1) denote a bulky organic cation, like [(n‑C_4_H_9_)_4_N]^+^ or [(C_6_H_5_)_4_P]^+^, and a weakly coordinating anion, like [Al(OC(CF_3_)_3_)_4_]^−^, respectively. The borohydride product (in this case CsY(BH_4_)_4_) is precipitated from the solvent like dichloromethane, leaving most contaminants in the remaining solution. The above synthetic method has been utilized for a number of products, containing various metal cations, including those belonging to the *s*- and *d*- blocks of periodic table, while among the *f*-elements only the borohydrides containing Eu^III^ have been attempted [37].

In this contribution we discuss synthesis, crystal structures, and thermal decomposition of a series of borohydrides of trivalent rare-earth (*RE*) elements: (n‑C_4_H_9_)_4_N*RE*(BH_4_)_4_, for *RE* = Ho, Tm and Yb. These compounds can be conveniently prepared and are thermally stable, therefore they may serve as precursors towards new mixed-cation borohydrides of the *RE* mentioned above, which would be performed according to Equation (1). Notably, only a limited number of inorganic, solvent-free borohydrides of *RE* = Ho, Tm, and Yb is currently known, including KHo(BH_4_)_4_ [38], RbTm(BH_4_)_4_ [23] and *M*Yb(BH_4_)_4_, where *M* = Li [11], Na, K [39]. It is worth to mention that although a few (n‑C_4_H_9_)N*RE*(BH_4_)_4_ compounds have been reported before [40], only two of them, *RE* = Y [41] and Sc [42], were fully identified on a basis of their crystal structures.

## 2. Materials and Methods

### 2.1. Reagents Handling

All reagents were handled in a glovebox under argon atmosphere (H_2_O < 1 ppm, O_2_ < 1 ppm). All chemicals were anhydrous, fine quality reagents of high purity (*RE*Cl_3_ > 99.9%; LiBH_4_ > 95%; (n‑C_4_H_9_)_4_N(BH_4_) (TBAB) >98%; n-hexane > 99%; dichloromethane (DCM) > 99.8%) purchased from Sigma Aldrich. Anhydrous DCM was additionally distilled with P_2_O_5_ and to some portions molecular sieves were added as well.

### 2.2. Synthesis

Mechanochemical reactions were carried out utilizing LMW–S vibrational mill (Testchem, 1400 rpm) and milling vessel made of silicon carbide. All millings were conducted in 5 min periods, with overall time of 60 min. Between these periods the vessel was cooled down using liquid nitrogen to maintain room temperature (RT), thereby avoiding thermal decomposition of the products. The vessels were sealed inside the glovebox filled with argon. The samples were synthesized using 1 mmol of *RE*Cl_3_, and other reagents, according to the following reaction scheme:

[(n-C_4_H_9_)_4_N]BH_4_ + *RE*Cl_3_ + 3 LiBH_4_ → [(n-C_4_H_9_)_4_N]*RE*(BH_4_)_4_ + 3 LiCl (2)

### 2.3. Powder X-ray Diffraction (PXRD)

PXRD measurements were performed using two diffractometers: Bruker D8 Discover and Empyrean series 2, both with parallel beam and the CuK_α1_ and CuK_α2_ radiation (intensity ratio of 2:1). Powder samples were sealed under argon atmosphere inside quartz capillaries (diameter of 0.3–0.5 mm).

### 2.4. Rietveld Refinement

Jana2006 program [43] was used for Rietveld refinement. [(n-C_4_H_9_)_4_N]Y(BH_4_)_4_ [41] and [(n-C_4_H_9_)_4_N]Sc(BH_4_)_4_ [42] were utilized as preliminary structural models for α-[(n-C_4_H_9_)_4_N]*RE*(BH_4_)_4_ and β-[(n-C_4_H_9_)_4_N]*RE*(BH_4_)_4_, respectively. Pseudo–Voight function was used for peak shape description, and Berar–Baldinozzi function or correction by divergence to describe their asymmetry. The background was described by 36 Legendre polynomials. A set of restrains was applied to keep suitable geometries. For BH_4_^−^ groups the B–H distances were fixed at 1.15 Å (with standard uncertainty, s.u. (standard uncertainty) equal to 0.001 Å) and H–B–H angles were fixed at 109.47° (s.u. = 0.01°). For [*RE*(BH_4_)_4_]^−^ a tridentate coordination scheme was set, where *RE*–H distances for the three H atoms from each borohydride group were fixed to be equal (s.u. = 0.01 Å). The Atomic Displacement Parameters (ADP) of hydrogen atoms were fixed as 1.2 ADP of boron atoms. If needed, inter H...H contact between TBA groups was restrain to at least 2 Å. For [n‑But_4_N]^−^ two approaches have been applied. For α-[(n‑C_4_H_9_)_4_N]*RE*(BH_4_)_4_ ADP for all B atoms were kept the same, as well as ADP for all C and N atoms. The C–H distances were restrained to 1 Å (s.u. = 0.001 Å), C–C and C–N distances to 1.5 Å (s.u. = 0.01 Å). Suitable C–C–C, C–N–C, N–C–C, N–C–H, C–C–H, and H–C–H angles were restrained to 109.47° (s.u. = 1°) to keep TBA^+^ geometry. ADPs of hydrogen atoms were fixed as 1.2 ADP of bonded C-atom. For β-[(n‑C_4_H_9_)_4_N]*RE*(BH_4_)_4_ (higher symmetry) suitable atoms were kept tetrahedral and ADP of hydrogen contained in organic cation as 1.2 or 1.5 ADP of the neighboring carbon for the middle and terminal carbon atom, respectively (riding model). 

### 2.5. Recrystallization of the Samples

Samples were recrystallized using DCM in which TBA*RE*B is very well soluble, while the insoluble LiCl can be removed easily using filtration or decantation. A simple, slow evaporation of the solvent at room temperature led to poor quality, highly mosaic, and twinned crystals. Much better results were obtained when the process of crystallization has been carried out via diffusion of hexane vapor to the DCM solution of TBA*RE*B.

### 2.6. Single-Crystal X-ray Diffraction (SC-XRD)

The crystals were measured on Agilent Supernova X-ray diffractometer with CuK_α_ micro-source. Data collection and reduction were performed with CrysAlisPro software (version 39.46, by Rigaku Oxford Diffraction) [44], while SHELXT [45] and Olex2 [46,47] programs were applied for structure solution and refinement, respectively.

### 2.7. The Cambridge Crystallographic Data Centre (CCDC)

α‑[(n‑C_4_H_9_)_4_N]*RE*(BH_4_)_4_, *RE* = Ho (PXRD: 2058633), Tm (PXRD: 2058634), and β‑[(n‑C_4_H_9_)_4_N]*RE*(BH_4_)_4_, *RE* = Ho (SC-XRD: 2058684 for 100 K, 2058683 for 200K and 2058678 for 300 K), Tm (PXRD: 2058972; SC-XRD: 2058681 for 100 K, 2058680 for 200 K, and 2058682 for 300 K), Yb (PXRD: 2058635; SC-XRD: 2058679 for 100 K), contain the supplementary crystallographic data for this paper. These data can be obtained free of charge via http://www.ccdc.cam.ac.uk/conts/retrieving.html or from the Cambridge Crystallographic Data Centre. Additionally, preliminary structure for α‑[(n‑C_4_H_9_)_4_N]Ho(BH_4_)_4_ from SC-XRD (100 K) is placed in S4 part of ESI, due to poor quality of obtained crystals.

The crystal structures were visualized using VESTA [48].

### 2.8. Thermal Decomposition: TGA/DSC/EGA

Samples were placed in Al_2_O_3_ crucibles with a small hole in the lid, allowing the gaseous thermal decomposition products to be analyzed. Powder samples were analyzed using Netzsch STA 409 PG instrument, utilizing combined thermogravimetry (TGA) and differential scanning calorimetry (DSC) simultaneous measurements. Measurements were performed in the range from room temperature (RT) to 650 °C at 5 °C/min heating rate, at a constant 100 mL/min flow of high-purity argon. The evolved gaseous products were transported through quartz capillary preheated to 200 °C to the quadrupole mass spectrometer (Netzsch / Pfeiffer Vacuum, model QMS 403C). After the measurements the samples were cooled down to RT in argon atmosphere. Then solid products of thermal decomposition were collected, put inside the glovebox and analyzed using PXRD. A short contact with air was not fully prevented during collection of solid decomposition products.

### 2.9. Spectral Analysis: FTIR

Spectra were collected in 4000–400 cm^−1^ range using Vertex 80v FTIR spectrometer from Bruker and anhydrous KBr (from Sigma Aldrich, 200 mg per pellet of 12 mm diameter) as pellets material.

## 3. Results and Discussion

### 3.1. Synthesis Outcome and Product Identification

**Figure 1:**
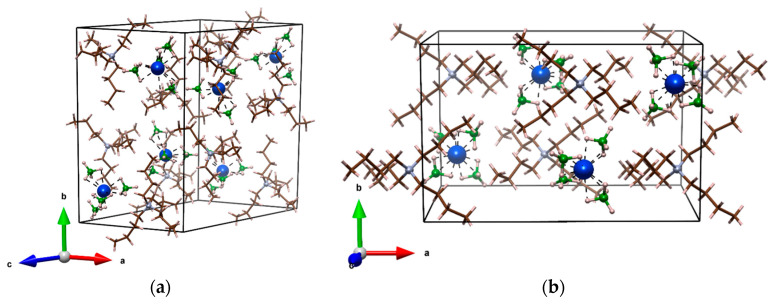
Crystal unit cells of: (**a**) α‑[(n‑C_4_H_9_)_4_N]*RE*(BH_4_)_4_ (here *RE* = Ho); (**b**) β‑[(n‑C_4_H_9_)_4_N]*RE*(BH_4_)_4_ (here *RE* = Ho). Blue color—*RE* atoms; green—B atoms; brown—C atoms; grey—N atoms; pink—H atoms.

A series of [(n‑C_4_H_9_)_4_N]*RE*(BH_4_)_4_ compounds, *RE* = Ho, Tm, Yb has been prepared according to Equation (2). The products obtained were identified by PXRD, and are summarized in Table 1. The prepared [(n‑C_4_H_9_)_4_N]*RE*(BH_4_)_4_ (abbreviated TBA*RE*B) crystalize in two different structure types observed previously for similar compounds: α- of TBAYB crystal structure (*P*2_1_/c) [41], and β-, isostructural to TBAScB (*P*nna) [42], cf. Figure 1. The polymorphs have been named in analogy to those of the single-cation rare earth borohydrides for which α- denotes a lower symmetry form [11,38,49,50,51,52,53]. For better structural description, the samples were recrystallized and single crystals were investigated by SC-XRD (see: Materials and Methods); the crystal structures have been discussed in the Section 3.2. The plots showing Rietveld analysis of the as-milled samples are placed in Supplementary Materials, Figures S1–S3.

The identity of polymorph dominating in the raw, post-reaction mixture (α- or β-) strongly correlates with *RE*^3+^ ionic radius. In the case of the largest Ho^3+^, revealing similar radius to Y^3+^, only α-TBAHoB phase (along with LiCl) can be detected, which reminds the behavior of TBAYB [41]. On the other hand, the smallest Yb^3+^ forms β-TBAYbB isostructural to β‑TBAScB [42]. In this case, the same polymorph is preferred despite the effective size of Yb^3+^ is ca. 15% larger than that of Sc^3+^, while there is only <4% difference between Yb^3+^ and Ho^3+^ [54]. For Tm^3+^ of intermediate size, both polymorphs, α‑TBATmB and β‑TBATmB, are present in a comparable amount, with the molar ratio of 1:0.96, respectively, according to Rietveld refinement (Figure S2).

Recrystallization of the as prepared TBAHoB and TBATmB via slow evaporation of DCM led to formation of α‑TBAHoB and β‑TBATmB, respectively, with traces of unidentified crystalline phase according to PXRD (Figures S4 and S5, respectively). However, the unidentified impurities contribute only to minor diffraction intensity, especially for *RE* = Tm. Unfortunately, it appeared that the crystals obtained this way revealed rather poor quality for *RE* = Ho and Tm (showing high mosaicity and severe twinning), while the crystals of sufficient quality for SC-diffraction measurements were obtained only for β‑TBAYbB. For *RE* = Tm and Ho much better single crystals were obtained via diffusion of hexane vapor to the DCM solution of TBA*RE*B, surprisingly resulting only in β‑TBA*RE*B in both cases. Apparently, the choice of solvent and the conditions of crystallization influence the polymorphs obtained, indicating their proximity in formation enthalpy.

### 3.2. Crystal Structures and Polymorphism of [(n‑C_4_H_9_)_4_N]RE(BH_4_)_4_

Crystal structures obtained from the diffraction measurements performed at RT, ca. 293 K (PXRD) and at controlled temperature of 100, 200, and 300 K (SC-XRD) for TBA*RE*B, *RE* = Y, Ho, Tm, Yb, Sc, are summarized and compared in Table 2 and Table S1 (in the Supplementary Materials).

α‑[(n‑C_4_H_9_)_4_N]*RE*(BH_4_)_4_ adopt monoclinic unit cell crystalizing in *P*2_1_/c centrosymmetric space group, presented at Figure 1a. This structure was previously described for α‑TBAYB analogue [41]. A comparison of the unit cells for *RE* = Ho, Y, Tm is presented in Table 2. It is worth noting, that β angle is similar for all those compounds (around 129°). β‑[(n‑C_4_H_9_)_4_N]*RE*(BH_4_)_4_ has a higher symmetry and adopt orthorhombic unit cell, space group *P*nna, seen at Figure 1b. It strongly resembles the crystal structure of TBAFeCl_4_ [55], where the chloride ions are replaced by the borohydride groups. This structure was previously described for β‑TBAScB analogue [42].

Both α‑TBA*RE*B and β‑TBA*RE*B polymorphs of these ionic compounds are composed of [(n‑C_4_H_9_)_4_N]^+^ cations (abbreviated TBA^+^) and [*RE*(BH_4_)_4_]^−^ anions, with Z’ = 1 and 0.5, respectively, Figure 1. In the first of them each TBA^+^ is surrounded by four [*RE*(BH_4_)_4_]^−^, forming a distorted tetrahedron, while [*RE*(BH_4_)_4_]^−^ is surrounded in a deformed trigonal pyramid pattern by the four nitrogen centers of TBA^+^ cations (Figure S6). In the case of β‑TBA*RE*B polymorph both TBA^+^ and [*RE*(BH_4_)_4_]^−^ have the same coordination spheres, with five counterions around them constituting deformed trigonal bipyramids. The latter form a three-dimensional network with N and *RE* atoms placed in a honeycomb-like structure, taking alternating positions in the corners of each hexagon (Figure S7).

In the case of β‑TBA*RE*B, *RE* = Sc [42], and described here by powder method for *RE* = Tm, Yb a better Rietveld fit for **Yb** has been acquired modeling slight disorder of the aliphatic chains in the TBA^+^ cation. However, such disordered model was not necessary for a reliable refinement of SC-XRD data for *RE* = Ho, Tm, Yb. The aliphatic chains present in both structures of TBA*RE*B are rather close to the idealized geometry. This was not the case for the parent TBAB showing significantly deformed and severely disordered TBA^+^ moieties already at 100 K [41].

In both cases *RE^3^*^+^ is coordinated by the four borohydride groups with boron centers forming a tetrahedron around *RE*. Although the positions of hydrogen atoms are significantly biased by their low scattering factor, the FTIR spectra (Figure S20 in ESI) show the features characteristic for BH_4_^−^ anions acting as tridentate ligands [56], which results in 12-fold coordination. The observed absorption bands are also similar to those reported for the related derivatives of yttrium borohydride [57], where the DFT calculations for [(CH_3_)_4_N][Y(BH_4_)_4_] confirmed 12-fold coordination of Y^3+^ in the [Y(BH_4_)_4_]^−^ complex. [*RE*(BH_4_)_4_]^−^ complex anion in such geometry is found in numerous *RE^3^*^+^ borohydrides, also in all the known mixed-cation derivatives of *RE*(BH_4_)_3_, *RE* = Ho, Tm, Yb, namely K[Ho(BH_4_)_4_] [38], Rb[Tm(BH_4_)_4_] [23], [Ph_4_P][Tm(BH_4_)_4_] [58], and *M*[Yb(BH_4_)_4_], *M* = Li [11], Na, K [39], as well as in the related compounds of Sc and Y [34,57,59,60].

**Figure 2:**
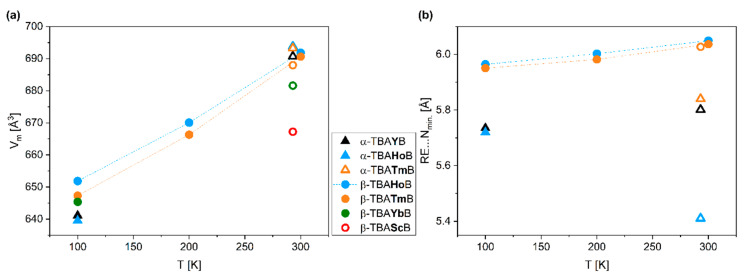
(**a**) Molecular volumes (V_m_), and (**b**) minimal RE…N distances in the function of temperature for a series of TBAREB compounds in both α– and β– forms. SC-XRD data—filled symbols, PXRD data—open symbols.

Under specified temperature conditions, the molecular volume (V_m_) of β-TBA*RE*B is related to *RE*^3+^ ionic radius, Table 2 and Table S1. At 100 K the α‑TBA*RE*B polymorph of TBAHoB reveals slightly smaller molecular volume (by ca. 2%, from SC-XRD data), while this trend inverts at RT—the molecular volume of α‑TBATmB is ca. 1% larger than that of β‑TBATmB, as judged from the PXRD data, Figure 2a. On the other hand, the minimal distances between the ion centers, as probed by the *RE*…N distances, are significantly shorter for α‑TBA*RE*B polymorph (*>*4%), regardless of the temperature, Figure 2b. This difference is even more distinct for TBAHoB, showing slightly different geometry of the TBA^+^ cation resulting in smaller ion separation, Figure S8.

### 3.3. Thermal Decomposition Process and Evolved Gas Analysis

**Figure 3:**
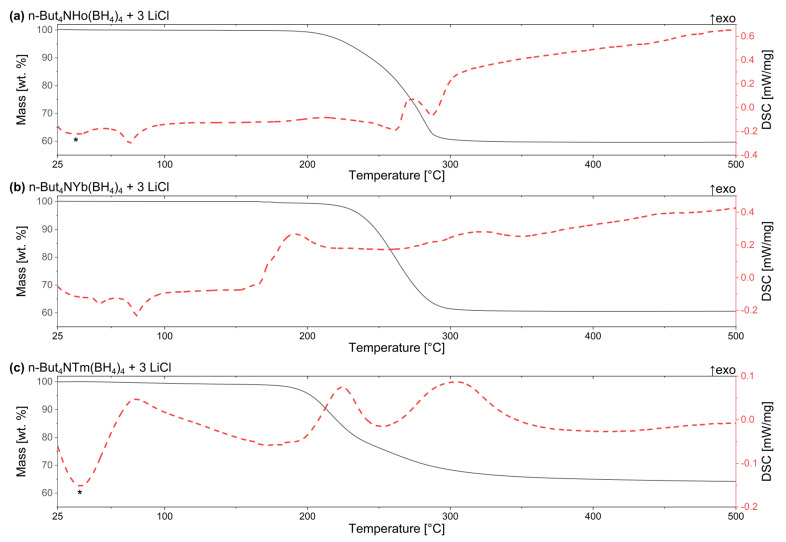
TGA/DSC curves of the crude, not purified samples (**a**) **Ho**, (**b**) **Yb,** and (**c**) **Tm**, prepared as in Table 1. *—artifact, coming from the instrument.

TGA curves for all the samples reveal major drop of mass between ca. 190 and 300 °C, indicating the main stages of thermal decomposition, Figure 3 and Figure S10–S16 in Supplementary Materials. For **Ho** and **Yb** samples (Table 1), thermal decomposition is preceded by melting around 75 and 80 °C, respectively, as judging from DSC extrema. In the case of **Yb** melting is preceded probably by a polymorphic transition revealing minor endothermic effect around 54 °C. This renders TBAHoB and TBAYbB ionic liquids, similar to their analogues described previously: TBAYB melting around 78 °C [41] and TBAScB melting at 88 °C [42]. In the case of **Tm** sample an exothermic event can be observed close to this temperature which is associated with a minor mass loss (<1 wt.%) due to emission of volatile products (Figures S12 and S15). The signals observed in the time-resolved mass spectra suggest emission of B_2_H_6_ during this decomposition stage, which could indicate partial reduction to Tm^2+^. This sample shows no signs of melting, as judged from lack of clear endothermic DSC signal and the physical appearance of the post-decomposition residue, which remains powder-like, contrary to the other two samples (see Section 3.4).

Around 175 °C, an intense evolution of H_2_ and B_2_H_6_ is observed on MS spectrum of **Yb** sample, along with an exothermic effect seen at DSC curve, and a minor mass loss (ca. 1.2 wt.%). This should be associated with Yb^3+^ → Yb^2+^ reduction which takes place in the case of other borohydrides containing ytterbium [11,50]. In this respect, TBAYbB is slightly more stable thermally in comparison to its inorganic analogues; a similar effect has been observed for the borohydrides of Eu^3+^ and Sm^3+^ [11,37,51,61]. 

According to temperature-resolved MS spectra, in the case of **Ho** and **Yb** samples volatile organic decomposition products are evolved within the range of 175–375 °C, along with the fragments of BH_4_ groups. The signals from hydrogen are present in the range of 250 to 325 °C and 225 to 650 °C for **Ho** and **Yb** sample, respectively. On the other hand, during thermal decomposition of **Tm** sample the MS signals from the volatile organic decomposition products are detected in the range of 135 to 500 °C, with an additional upcast of signals just before 600 °C. In this case, strong signals from hydrogen are observed starting from 175 °C till the end of the measurement.

Above 600 °C the melting of LiCl is observed at DSC curves of all the samples, with much smaller effect seen for **Yb**. Such weakening of the thermal effect may be caused by partial consumption of LiCl due to formation of a halide-substituted derivative, as it has been observed during heating for inorganic borohydrides of ytterbium and scandium [50,62] and in as-milled samples of various others lanthanides borohydrides [51,63,64,65]. The total mass drop at TGA curves for **Ho**, **Tm** and **Yb** samples is 40.2, 36.6, and 39.5%, respectively. As the LiCl does not contribute to the drop of mass, it can be associated only with thermal decomposition of TBA*RE*B, which leads to the corrected mass drops of 51.2, 46.5, and 50.1%, respectively. Thermal decomposition processes discussed here for TBA*RE*B, *RE* = Ho, Tm, Yb, are similar to those observed for RE = Y [41], Sc [42]. TBAYB and TBAScB decompose endothermally, above 230 and 175 °C, respectively. Evolution of volatile organic decomposition products in the case of yttrium and scandium analogs leads to ca. 60% (corrected for pure compound) and 64% mass drop, respectively. These complex borohydrides are usually more stable thermally in comparison to organic (*n*‑C_4_H_9_)_4_NBH_4_, which melts around 130 °C and its exothermic decomposition starts above 160 °C with a mass drop around 87.5% [41].

### 3.4. Analysis of Solid Residues from Thermal Decomposition 

The process of thermal decomposition of borohydrides may be utilized for synthetic purposes. The recent studies show that lanthanide borohydrides can serve as precursors of crystalline lanthanide borides [11,51], which can be prepared via annealing of the former compounds to 650 °C. What is more, thermal decomposition of (NH_4_)_3_Mg(BH_4_)_5_ was reported to led to formation of amorphous BN, which can be obtained in the pure form after rinsing the product with water [16]. These findings encouraged us to analyze the chemical composition of the residue left after heating of TBA*RE*B to 650 °C. The crystalline phases detected by PXRD are summarized in Table 1.

In the case of **Ho** and **Yb** samples the residue is embedded in the crucible, and also on the top of the lid, which suggests melting and boiling of the samples and blending into the crucible pores during the process in which the residue is formed. Contrary, the post-decomposition residue of the **Tm** sample remains in the form of a pellet, and it is not spread on the top of the lid and it is not blended into the crucible, resembling the form of residue found after thermal decomposition of simple lanthanide borohydrides [11]. It appeared that the respective oxides and borates are the only *RE*-containing crystalline phases detected in the decomposed samples instead of the lanthanide borides (which should also be stable during short contact with air). Similar crystalline oxide-containing phases were detected for several samples of decomposed inorganic borohydrides of *RE*(BH_4_)_3_, which indicated formation of the active *RE*H_x_, instantly reacting with atmospheric O_2_/H_2_O [11]. Note that the contact with air was possible during the transportation of the crucibles (containing residue) to the glovebox. In all the samples the respective *RE*BO_3_ have been observed in crystalline form, along with remaining LiCl. Additionally, in the case of *RE* = Yb, Yb_2_O_3_ was observed, and in the case of *RE* = Ho additional signals were seen at the PXRD pattern, which can be assigned to Li_3_B_14_ and to a phase isostructural to Li_2_Yb_5_O_4_(BO_3_)_3_ [66], probably of a formula of Li_2_Ho_5_O_4_(BO_3_)_3_ (Figures S17–S19, structures from [66,67,68,69,70]). Unfortunately, the quality of PXRD pattern obtained does not allow for a reliable refinement of the latter structure. In the case of *RE* = Tm only TmBO_3_ and LiCl are present as crystalline phases according to the PXRD results.

## 4. Conclusions

Successful synthesis of three novel compounds, namely *n*‑But_4_N*RE*(BH_4_)_4_, *RE* = Ho, Tm, Yb, is reported, alongside with their crystal structures, polymorphism, and thermal properties. TBA*RE*B are synthesized using mechanochemical milling and are purified (from LiCl byproduct) via extraction with DCM. SC-XRD and PXRD studies show that they crystalize in a monoclinic (*P*2_1_/c, α-TBA*RE*B) or an orthorhombic (*P*nna, β-TBA*RE*B) crystal system. The former was found in the as-milled TBA*RE*B samples, *RE* = Ho, Tm, and in the TBAHoB samples recrystallized in DCM, while the latter in the as-milled and crystallized in DCM TBA*RE*B samples for *RE* = Yb, Tm and in the TBA*RE*B samples, *RE* = Ho, Tm, when a mixture of DCM and hexane has been utilized for crystallization.

Unit cells of both polymorphs have similar volumes, β-TBA*RE*B form being less than 1% smaller at RT according to PXRD data, while the trend is being inverted at 100 K, where α-TBA*RE*B unit cell has smaller molecular volume by around 2%, as judged from SC‑XRD data. Size of the unit cell in the case of all *RE* increases with rising *RE*^3+^ ionic radius for β-TBA*RE*B polymorphs under specified temperature conditions, and also grows with rising temperature for both polymorphs. We found out that the minimal distances between the ion centers, probed by the *RE*…N distances, are significantly shorter for α-TBA*RE*B polymorph (>4%), while the difference is even more distinct for TBAHoB.

TBAHoB and TBAYbB melt around 75 °C, which renders them new ionic liquids. Such molten salts can be used in synthesis of other compounds in a reductive environment, extending the current utility of the ionic liquids with incorporated rare earth elements [71]. n‑But_4_N*RE*(BH_4_)_4_, *RE* = Ho, Tm, Yb, decompose with evolution of organic compounds, diborane, and hydrogen. Additionally, a reduction in Yb^3+^ → Yb^2+^ is observed with simultaneous intense evolution of diborane and hydrogen. Solid decomposition products after heating at 650 °C and short contact with air at RT consist of the corresponding *RE* boranes, oxides and lithium borides.

As the compounds obtained are soluble in the weakly basic solvents, they can be used as precursors of various inorganic mixed-cation lanthanide borohydrides, which synthesis in a pure form would be possible employing metathetic synthesis protocol of mixed-metal borohydrides [14,31,32,37].

## Figures and Tables

**Table 1:** Summary of the reactions of type (2) performed in this work, their conditions and crystalline products after the synthesis and after heating them to 650 °C (and short contact with air^1^ in the last case).

Sample Name	Substrates	Milling Time	Crystalline Products (Synthesis)	Crystalline Products (Heating to 650 °C)
**Ho**	HoCl_3_ + 3 LiBH_4_ + TBAB	12 × 5 min	α‑TBAHo(BH_4_)_4_, LiCl	HoBO_3_, LiCl, Li_3_B_14_, Li_2_Ho_5_O_4_(BO_3_)_3_
**Tm**	TmCl_3_ + 3 LiBH_4_ + TBAB	12 × 5 min	α‑TBATm(BH_4_)_4_, β‑TBATm(BH_4_)_4_, LiCl	TmBO_3_, LiCl
**Yb**	YbCl_3_ + 3 LiBH_4_ + TBAB	12 × 5 min	β‑TBAYb(BH_4_)_4_, LiCl	YbBO_3_, LiCl, Yb_2_O_3_

^1^ lid of a crucible has a little hole in the middle, allowing brief contact with air during transportation from TGA/DSC instrument to glovebox.

**Table 2:** Unit cell dimensions for TBA*RE*B, *RE* = Y, Ho, Tm, Yb, Sc. Data for 300 K were obtained from SC-XRD, data for RT (ca. 293 K) were obtained from PXRD.

Compound	α-TBAHoB	α-TBAYB [41]	α-TBATmB	β-TBAHoB	β-TBATmB	β-TBAYbB	β-TBAScB [42]
*RE^3+^* r ^1^ [Å]	0.901	0.900	0.880	0.901	0.880	0.868	0.745
spc. group	*P*2_1_/c	*P*2_1_/c	*P*2_1_/c	*P*nna	*P*nna	*P*nna	*P*nna
T [K]	RT	RT	RT	300	RT	RT	RT
a [Å]	11.4218(9)	11.4181(10)	11.4063(18)	19.3238(12)	19.287(3)	19.2235(10)	19.1399(10)
b [Å]	20.553(2)	20.510(3)	20.545(4)	12.0529(5)	12.0317(17)	11.9943(6)	11.8849(6)
c [Å]	15.3049(17)	15.2811(19)	15.319(3)	11.8820(6)	11.8591(19)	11.8244(6)	11.7325(6)
β [°]	129.433(7)	129.464(8)	129.423(12)	90	90	90	90
V [Å^3^]	2775.0(5)	2762.77	2773.1(10)	2767.4(3)	2751.9(7)	2726.4(2)	2668.9(2)
Z	4	4	4	4	4	4	4
V_m_ [Å^3^]	693.8	690.7	693.3	691.9	688.0	682.3	667.2

^1^ Effective ionic radius (6-coordinate, octahedral environment) from [54].

## Data Availability

Crystallographic data can be obtained free of charge via http://www.ccdc.cam.ac.uk/conts/retrieving.html or from the Cambridge Crystallographic Data Centre. For details see Section 2.7.

## Supplementary Materials

The following are available online at https://www.mdpi.com/1996-1944/14/6/1329/s1, Figure S1. Rietveld refinement for as-milled sample **Ho**, Figure S2. Rietveld refinement for as-milled sample **Tm**, Figure S3. Rietveld refinement for as-milled sample **Yb**, Figure S4. PXRD patterns with subtracted backgrounds for as-milled Ho sample (containing LiCl), purified Ho (with unknown impurities) and simulated pattern of α TBAHoB, Figure S5. PXRD patterns with subtracted backgrounds for as-milled **Tm** sample (containing LiCl), purified **Tm** (with unknown impurities) and simulated patterns of α‑TBATmB and β‑TBATmB, Table S1, supplementary to Table 2. Unit cell dimensions for TBA*RE*B, *RE* = Y, Ho, Tm, Yb, Sc. 100/200/300 K obtained from SC-XRD, RT from PXRD, Figure S6. Nitrogen centers of TBA^+^ cations and *RE*^3+^ in α‑TBA*RE*B, Figure S7. Honeycomb-like structure of nitrogen centers of TBA^+^ cations and *RE*^3+^ in β‑TBA*RE*B, Figure S8. *RE*…N distances for α‑TBAHoB and α‑TBATmB, Figure S9. Evolution of *a*, *b*, and *c* lattice parameters of β‑TBAHoB and β‑TBATmB in the function of temperature. SC-XRD data, Figure S10. TGA/DSC curves of the samples (a) **Ho**, (b) **Yb** and (c) **Tm**, up to 650 °C, Figure S11. MS spectrum for **Ho** sample, Figure S12. MS spectrum for **Tm** sample, Figure S13. MS spectrum for **Yb** sample, Figure S14. TGA/DSC curves for **Ho** sample, Figure S15. TGA/DSC curves for **Tm** sample, Figure S16. TGA/DSC curves for **Yb** sample, Figure S17. PXRD pattern (with subtracted background) of Ho sample heated to 650 °C, and simulated patterns of identified crystalline pyrolysis products, Figure S18. PXRD pattern (with subtracted background) of **Tm** sample heated to 650 °C, and simulated patterns of identified crystalline pyrolysis products, Figure S19. PXRD pattern (with subtracted background) of **Yb** sample heated to 650 °C, and simulated patterns of identified crystalline pyrolysis products, Figure S20. FTIR spectra of **Ho, Yb,** and **Tm** samples, and preliminary CIF of α-TBAHoB from SC-XRD data (100 K) in part S4.
