# Supplementary material for: Synthesis, Polymorphism and Thermal Decomposition Process of (n-C4H9)4NRE(BH4)4 for RE = Ho, Tm and Yb
Source: Materials (Basel). 2021 Mar 10;14(6):1329. doi: 10.3390/ma14061329 (PMC7999646; doi:10.3390/ma14061329)
Supplement: Supplementary file 1 [file materials-14-01329-s001.pdf]

materials

*Supplementary Materials*

# Synthesis, polymorphism and thermal decomposition process of (n-$C_4H_9$)$_4$N*RE*($BH_4$)$_4$ for *RE* = Ho, Tm and Yb

**Wojciech Wegner [1,2,*] and Tomasz Jaroń [2,*]**

[1] College of Inter-Faculty Individual Studies in Mathematics and Natural Sciences, University of Warsaw, Banacha 2c, 02-097 Warsaw, Poland
[2] Centre of New Technologies, University of Warsaw, Banacha 2c, 02-097 Warsaw, Poland
* Correspondence: w.wegner@cent.uw.edu.pl (W.W.), t.jaron@cent.uw.edu.pl. (T.J.)

## Contents

1. PXRD patterns, Rietveld refinement and structural information ........ 2
2. Thermal decomposition ........ 4
3. Solid decomposition products ........ 15
4. Preliminary CIF of α-TBAHoB from SC-XRD data (100 K) ........ 17
5. FTIR spectra of as-milled samples. ........ 22

**Citation:** Wegner, W.; Jaroń, T.; Synthesis, Polymorphism and Thermal Decomposition Process of (n-$C_4H_9$)$_4$N*RE*($BH_4$)$_4$ for *RE* = Ho, Tm and Yb. *Materials* **2021**, *14*, 1329. https://doi.org/10.3390/ma14061329

Academic Editor: Jacques Huot

Received: 27 January 2021
Accepted: 4 March 2021
Published: 10 March 2021

**Publisher's Note:** MDPI stays neutral with regard to jurisdictional claims in published maps and institutional affiliations.

**Copyright:** © 2021 by the authors. Submitted for possible open access publication under the terms and conditions of the Creative Commons Attribution (CC BY) license (http://creativecommons.org/licenses/by/4.0/).

## 1. PXRD Patterns, Rietveld Refinement and Structural Information

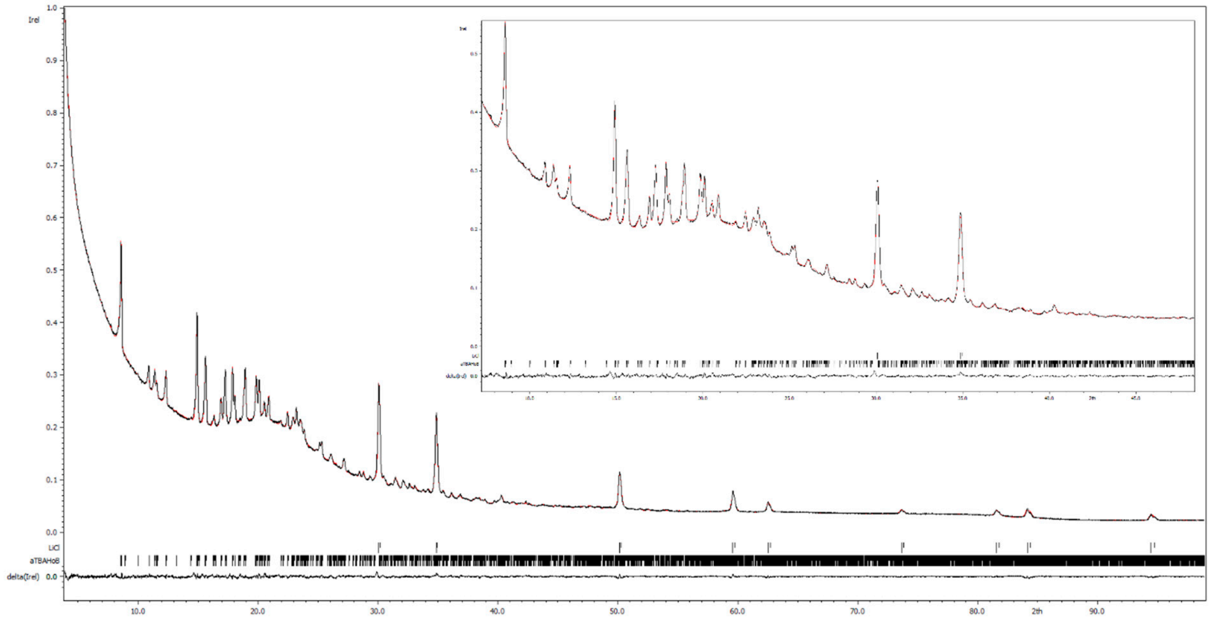


**Figure S1.** Rietveld refinement for as-milled sample **Ho**. Black curve represents experimental data, red curve – calculated profile. The position of the Bragg reflections has been marked and the difference curve (between the experimental and calculated profiles) are plotted at the bottom. Inset: the low angle region. GOF = 1.73; Rp = 0.73; wRp = 1.06. Wavelength: Cu ($K_{\alpha1}$ and $K_{\alpha2}$). Bragg reflections marked, from top to bottom, for: LiCl and $\alpha$-TBAHoB.

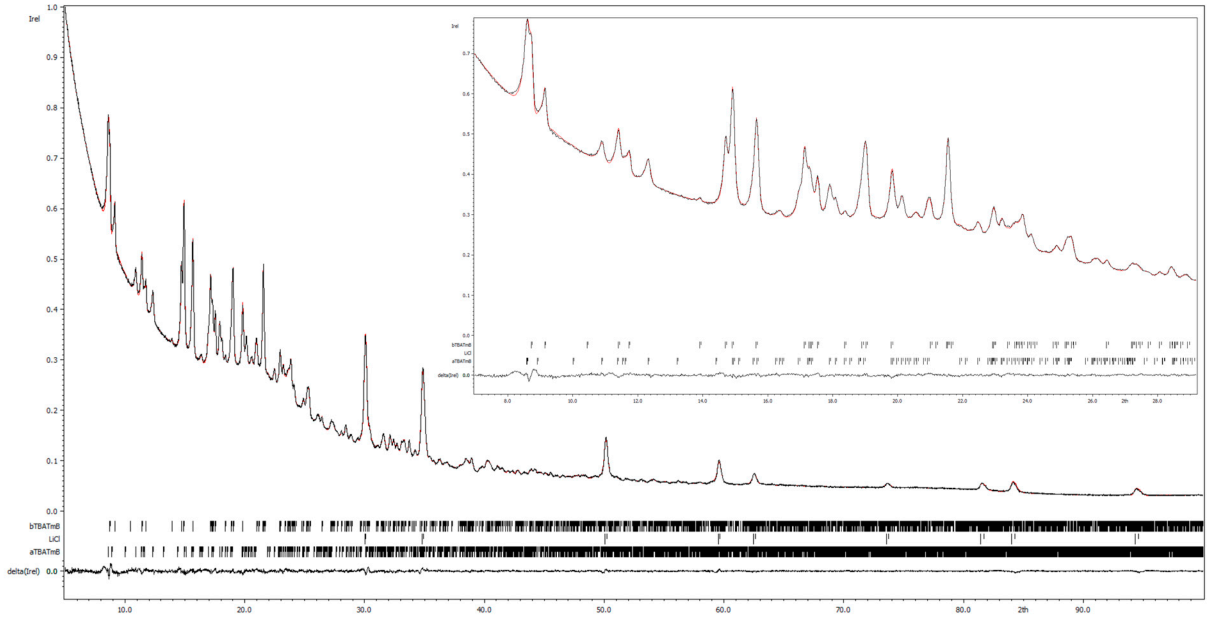


**Figure S2.** Rietveld refinement for as-milled sample **Tm**. Black curve represents experimental data, red curve – calculated profile. The position of the Bragg reflections has been marked and the difference curve (between the experimental and calculated profiles) are plotted at the bottom. Inset: the low angle region. GOF = 1.64; Rp = 0.69; wRp = 0.97. Wavelength: Cu ($K_{\alpha1}$ and $K_{\alpha2}$). Bragg reflections marked, from top to bottom, for: $\beta$-TBATmB, LiCl and $\alpha$-TBATmB.

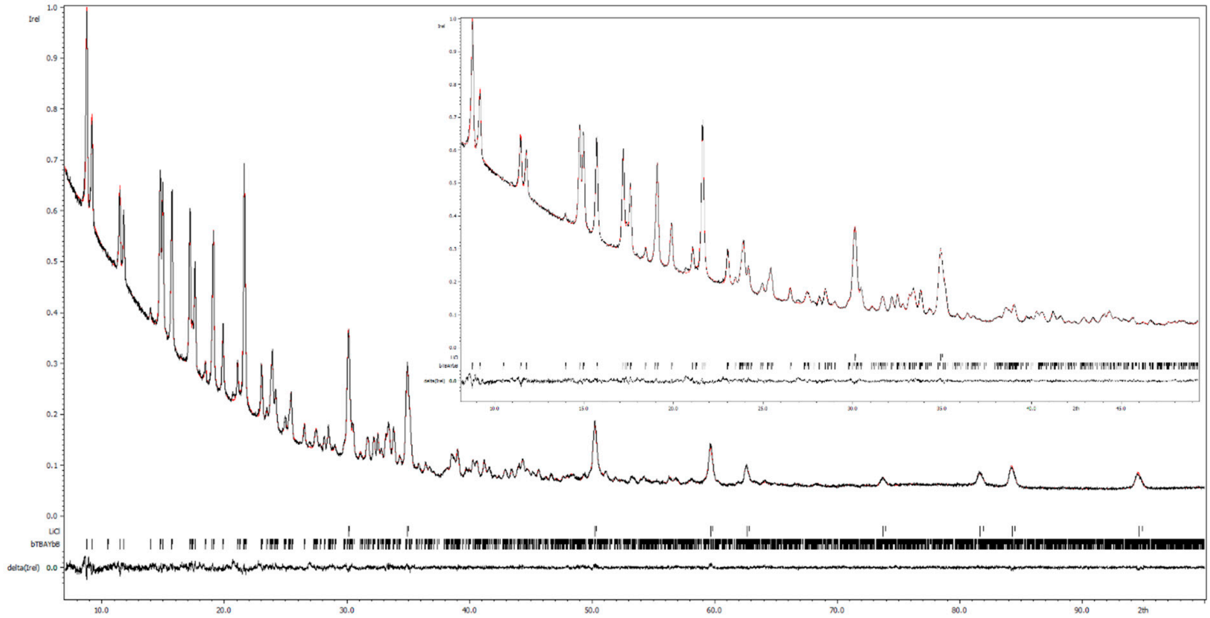


**Figure S3.** Rietveld refinement for as-milled sample **Yb**. Black curve represents experimental data, red curve – calculated profile. The position of the Bragg reflections has been marked and the difference curve (between the experimental and calculated profiles) are plotted at the bottom. Inset: the low angle region. GOF = 0.03; Rp = 1.29; wRp = 1.71. Wavelength: Cu ($K_{\alpha 1}$ and $K_{\alpha 2}$). Bragg reflections marked, from top to bottom, for: LiCl and β-TBAYbB.

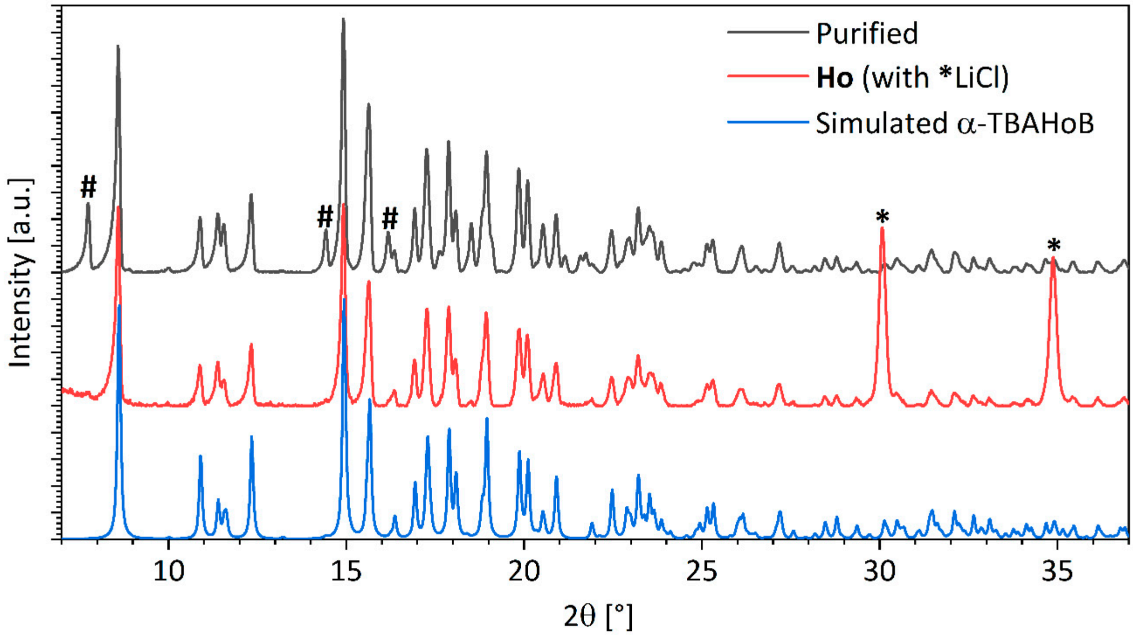


**Figure S4.** PXRD patterns with subtracted backgrounds for as-milled **Ho** sample (containing LiCl, marked *), purified **Ho** (with unknown impurities, marked #) and simulated pattern of α-TBAHoB (from cif, crystal structure obtained from Rietvield refinement).

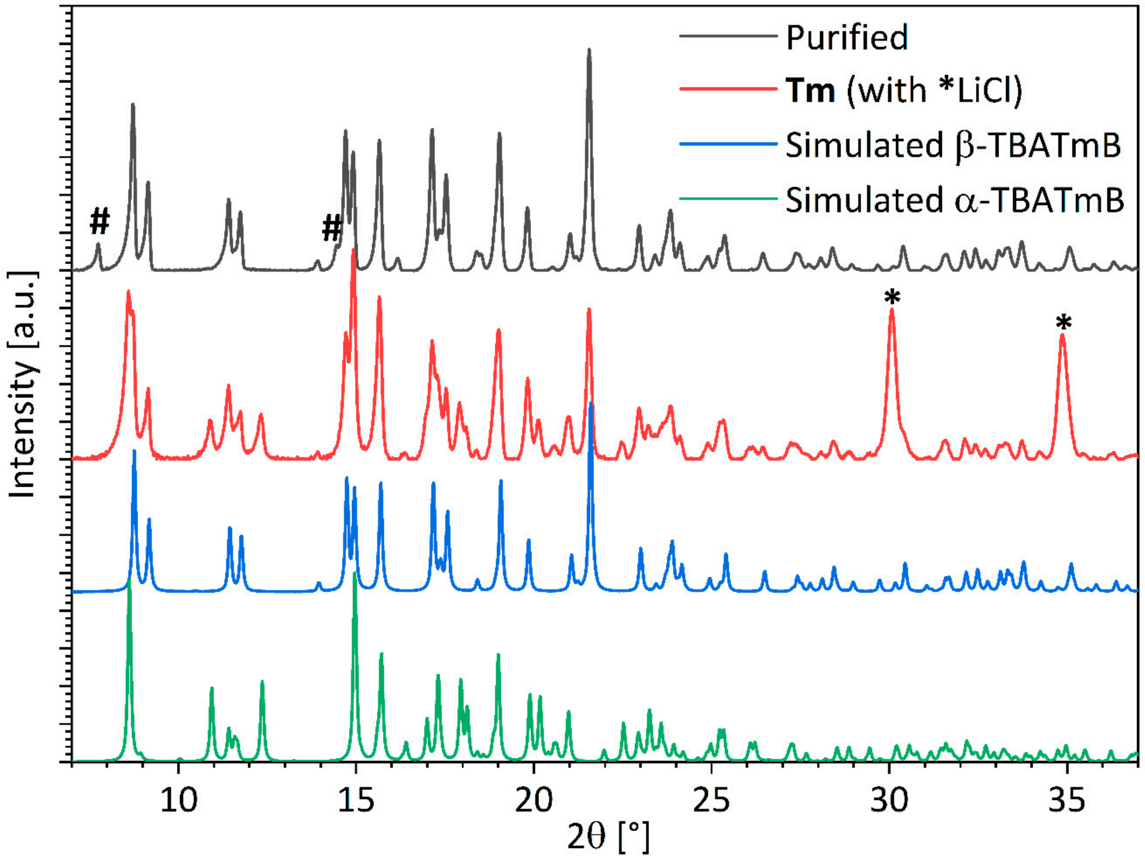


**Figure S5.** PXRD patterns with subtracted backgrounds for as-milled **Tm** sample (containing LiCl, marked *), purified **Tm** (with unknown impurities, marked #) and simulated patterns of α-TBATmB and β-TBATmB (from cifs, crystal structures obtained from Rietvield refinement).

**Table S1.** supplementary to **Table 2**. Unit cell dimensions for TBA*RE*B, *RE* = Y, Ho, Tm, Yb, Sc. 100/200/300 K obtained from SC-XRD, RT from PXRD.

| **Compd.** | **α-TBAYB [41]** | | **α-TBAHoB** | | **α-TBATmB** | **β-TBAHoB** | | | **β-TBATmB** | | | | **β-TBAYbB** | | **β-TBAScB [42]** |
|---|---|---|---|---|---|---|---|---|---|---|---|---|---|---|---|
| $RE^{3+}$ r[1] [Å] | 0.900 | | 0.901 | | 0.880 | 0.901 | | | 0.880 | | | | 0.868 | | 0.745 |
| spc. group | $P2_1/c$ | | $P2_1/c$ | | $P2_1/c$ | *P*nna | | | *P*nna | | | | *P*nna | | *P*nna |
| T [K] | 100 | RT | 100 | RT | RT | 100 | 200 | 300 | 100 | 200 | 300 | RT | 100 | RT | RT |
| a [Å] | 11.0453(5) | 11.4181(10) | 11.039(3) | 11.4218(9) | 11.4063(18) | 18.5597(8) | 18.9387(15) | 19.3238(12) | 18.5303(5) | 18.9730(6) | 19.3409(16) | 19.287(3) | 18.5673(9) | 19.2235(10) | 19.1399(10) |
| b [Å] | 20.0099(9) | 20.510(3) | 19.999(2) | 20.553(2) | 20.545(4) | 11.9188(3) | 11.9783(8) | 12.0529(5) | 11.8902(4) | 11.9295(4) | 12.0279(11) | 12.0317(17) | 11.8735(5) | 11.9943(6) | 11.8849(6) |
| c [Å] | 14.7204(8) | 15.2811(19) | 14.708(4) | 15.3049(17) | 15.319(3) | 11.7871(4) | 11.8153(9) | 11.8820(6) | 11.7514(3) | 11.7754(4) | 11.8749(12) | 11.8591(19) | 11.7096(5) | 11.8244(6) | 11.7325(6) |
| β [°] | 127.980(5) | 129.464(8) | 128.02(4) | 129.433(7) | 129.423(12) | 90 | 90 | 90 | 90 | 90 | 90 | 90 | 90 | 90 | 90 |
| V [Å$^3$] | 2564.44 | 2762.77 | 2558.1(10) | 2775.0(5) | 2773.1(10) | 2607.42(16) | 2680.3(3) | 2767.4(3) | 2589.17(13) | 2665.23(15) | 2762.5(4) | 2751.9(7) | 2581.5(2) | 2726.4(2) | 2668.9(2) |
| Z | 4 | | 4 | | 4 | 4 | | | 4 | | | | 4 | | 4 |

[1] Effective ionic radius (6-coordinate, octahedral environment) from [54]


| **Compd.** | **α-TBAYB [41]** | | **α-TBAHoB** | | **α-TBATmB** |
|---|---|---|---|---|---|
| $RE^{3+}$ r[1] [Å] | 0.900 | | 0.901 | | 0.880 |
| spc. group | $P2_1/c$ | | $P2_1/c$ | | $P2_1/c$ |
| T [K] | 100 | RT | 100 | RT | RT |
| a [Å] | 11.0453(5) | 11.4181(10) | 11.039(3) | 11.4218(9) | 11.4063(18) |
| b [Å] | 20.0099(9) | 20.510(3) | 19.999(2) | 20.553(2) | 20.545(4) |
| c [Å] | 14.7204(8) | 15.2811(19) | 14.708(4) | 15.3049(17) | 15.319(3) |
| β [°] | 127.980(5) | 129.464(8) | 128.02(4) | 129.433(7) | 129.423(12) |
| V [Å$^3$] | 2564.44 | 2762.77 | 2558.1(10) | 2775.0(5) | 2773.1(10) |
| Z | 4 | | 4 | | 4 |

| **Compd.** | **β-TBAHoB** | | | **β-TBATmB** | | | | **β-TBAYbB** | | **β-TBAScB [42]** |
|---|---|---|---|---|---|---|---|---|---|---|
| $RE^{3+}$ r[1] [Å] | 0.901 | | | 0.880 | | | | 0.868 | | 0.745 |
| spc. group | *P*nna | | | *P*nna | | | | *P*nna | | *P*nna |
| T [K] | 100 | 200 | 300 | 100 | 200 | 300 | RT | 100 | RT | RT |
| a [Å] | 18.5597(8) | 18.9387(15) | 19.3238(12) | 18.5303(5) | 18.9730(6) | 19.3409(16) | 19.287(3) | 18.5673(9) | 19.2235(10) | 19.1399(10) |
| b [Å] | 11.9188(3) | 11.9783(8) | 12.0529(5) | 11.8902(4) | 11.9295(4) | 12.0279(11) | 12.0317(17) | 11.8735(5) | 11.9943(6) | 11.8849(6) |
| c [Å] | 11.7871(4) | 11.8153(9) | 11.8820(6) | 11.7514(3) | 11.7754(4) | 11.8749(12) | 11.8591(19) | 11.7096(5) | 11.8244(6) | 11.7325(6) |
| β [°] | 90 | 90 | 90 | 90 | 90 | 90 | 90 | 90 | 90 | 90 |
| V [Å$^3$] | 2607.42(16) | 2680.3(3) | 2767.4(3) | 2589.17(13) | 2665.23(15) | 2762.5(4) | 2751.9(7) | 2581.5(2) | 2726.4(2) | 2668.9(2) |
| Z | 4 | | | 4 | | | | 4 | | 4 |

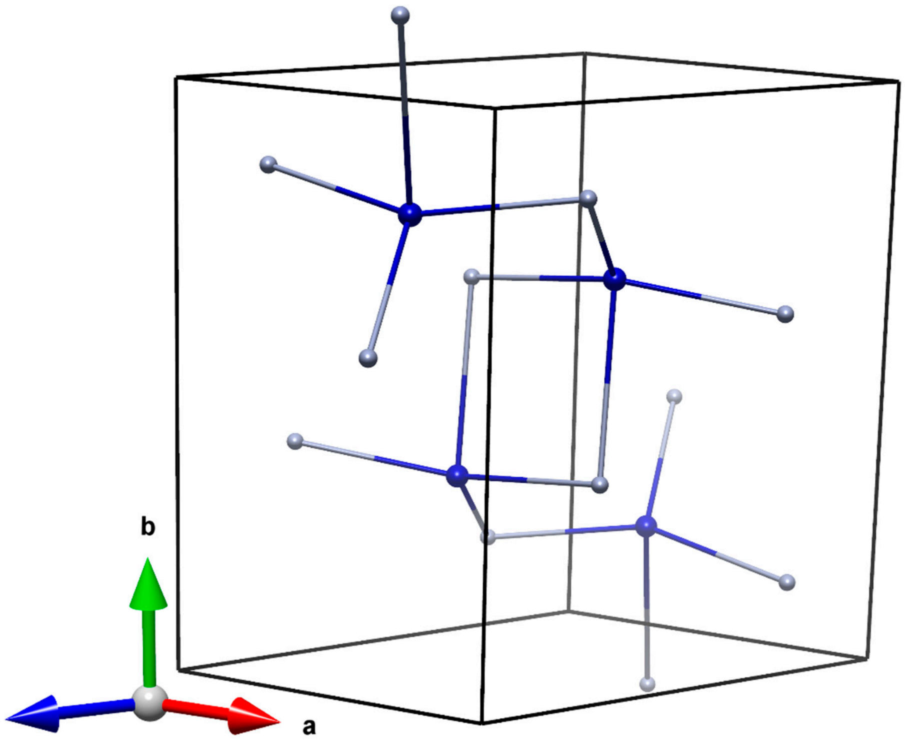


**Figure S6.** Nitrogen centers of TBA⁺ cations (marked gray) and $RE^{3+}$ (here *RE* = Tm, marked blue) in $\alpha$-TBA*RE*B.

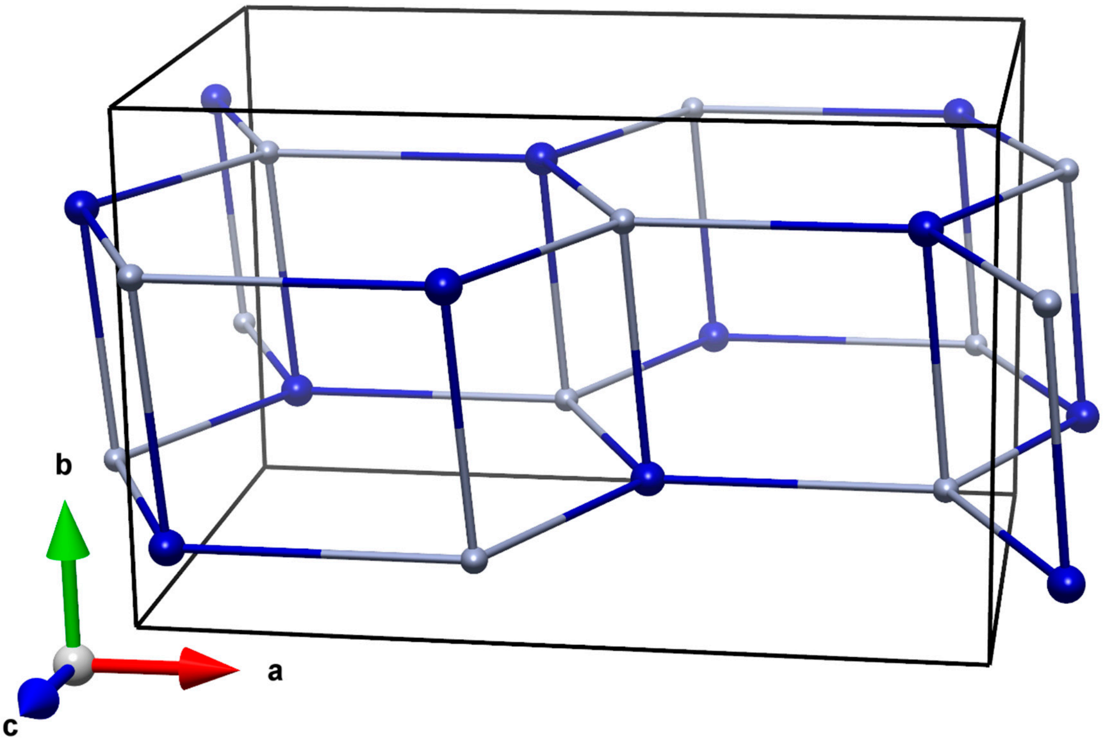


**Figure S7.** Honeycomb-like structure of nitrogen centers of TBA⁺ cations (marked gray) and $RE^{3+}$ (here *RE* = Tm, marked blue) in β-TBA*RE*B.

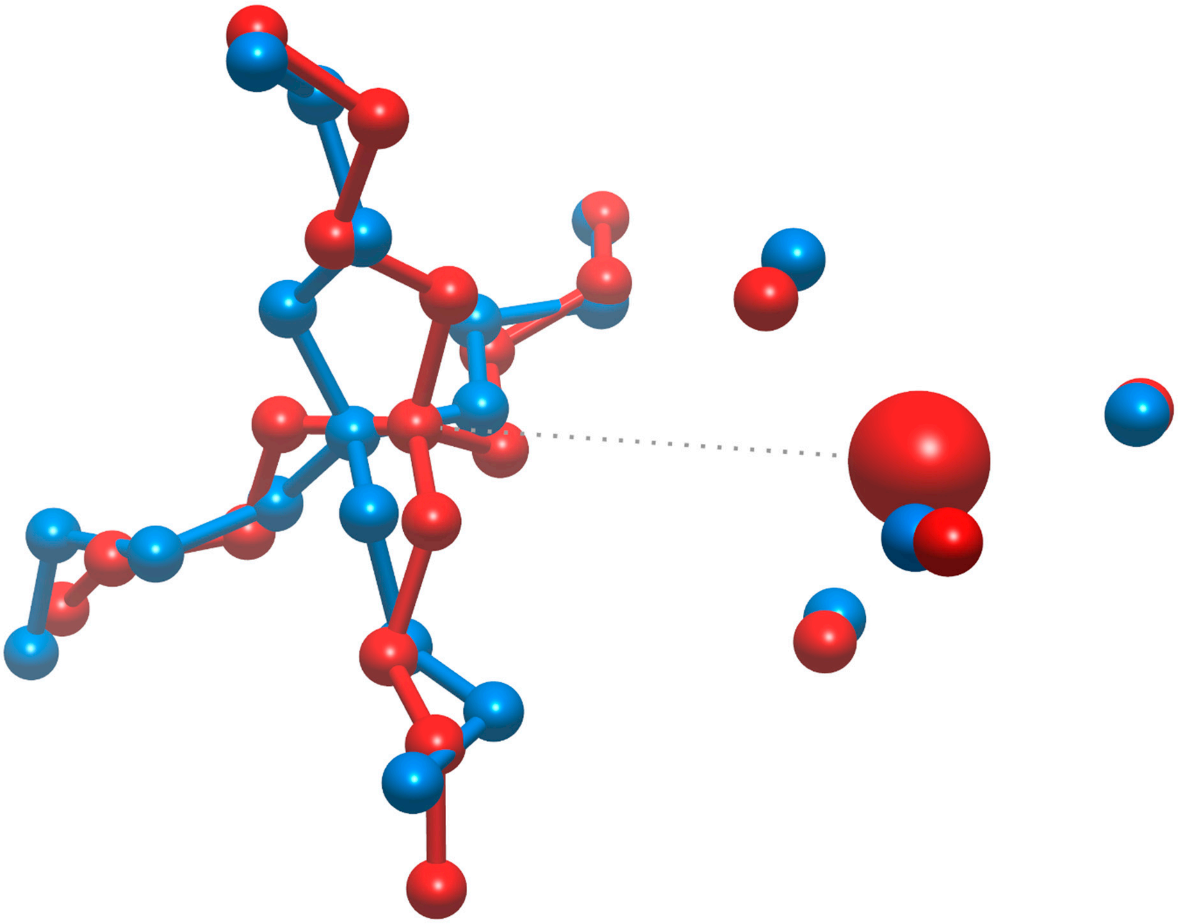

**Figure S8.** *RE*…N distances for α-TBAHoB (atoms marked red) and α-TBATmB (atoms marked blue), crystal structures obtained from PXRD. Left: $TBA^+$, right: $[RE(BH_4)_4]^-$. H atoms are not included.

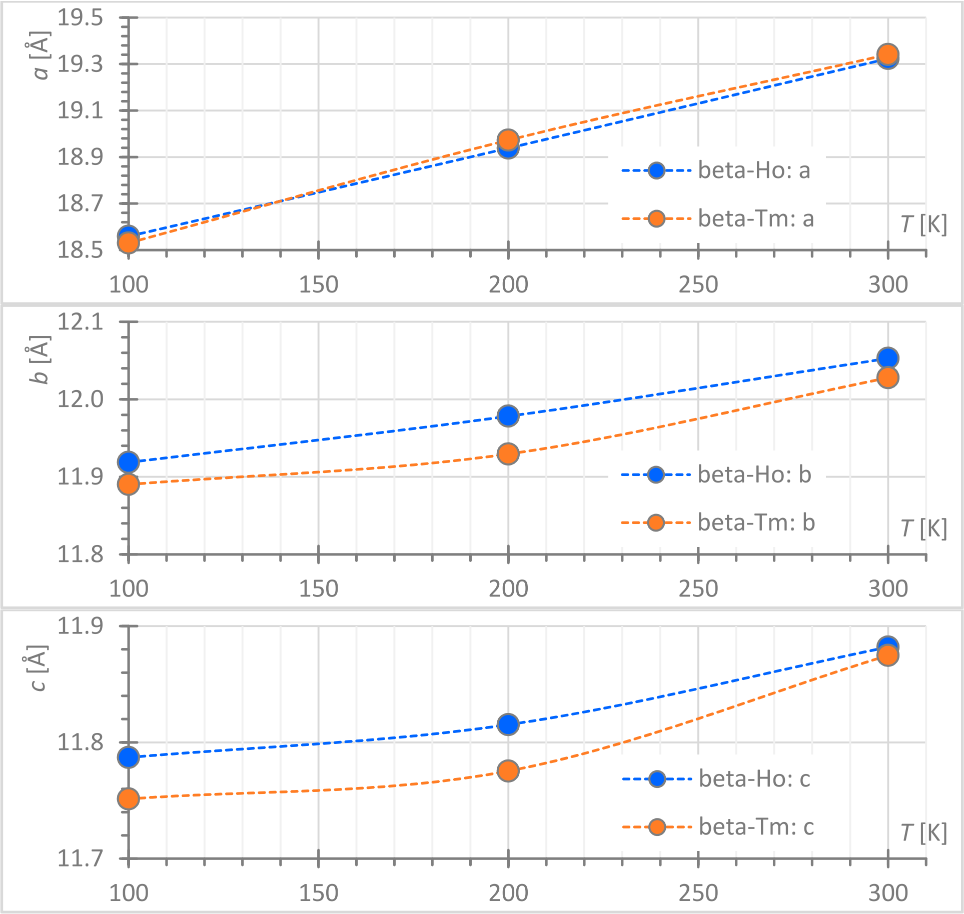


**Figure S9.** Evolution of *a*, *b*, and *c* lattice parameters of β-TBAHoB and β-TBATmB in the function of temperature. SC-XRD data.

## 2. Thermal decomposition

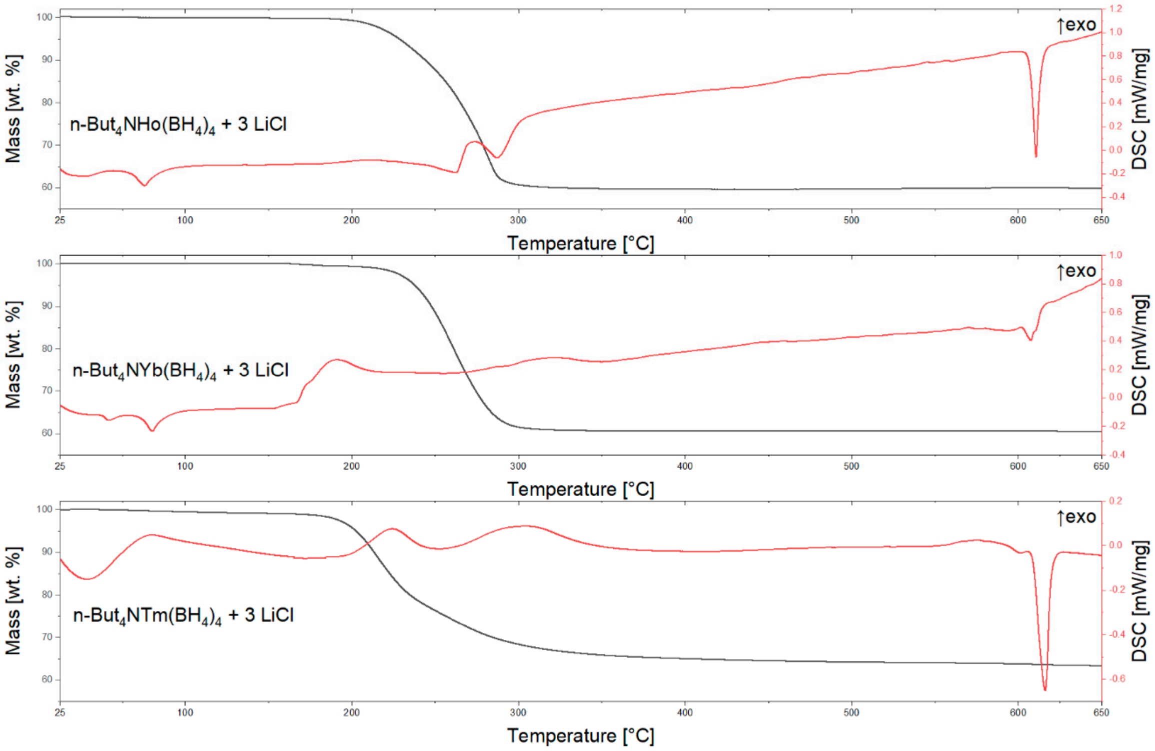


**Figure S10.** TGA/DSC curves of the as-milled samples (a) **Ho**, (b) **Yb** and (c) **Tm**, up to 650 °C.

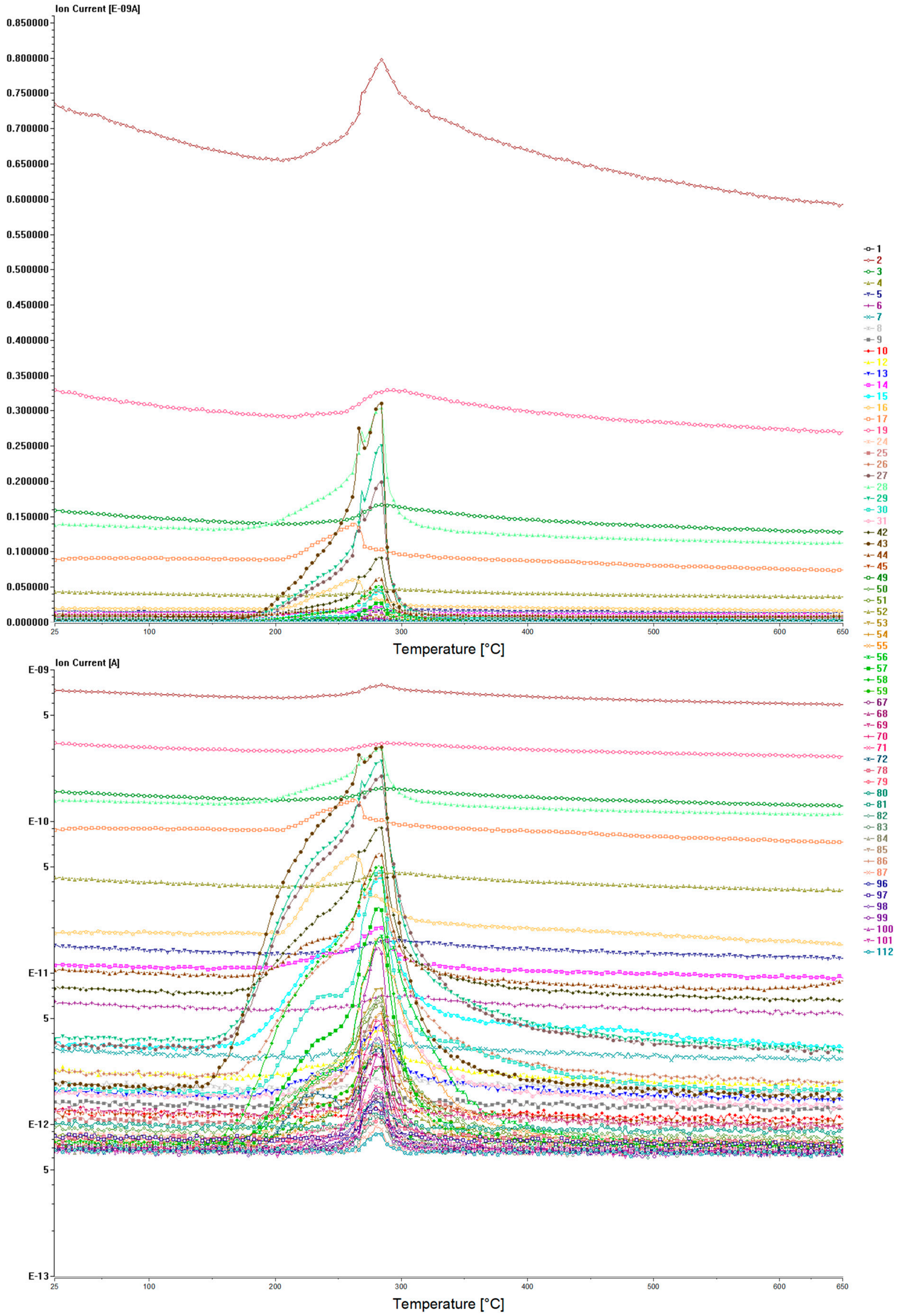


**Figure S11.** MS spectrum for **Ho** sample. Top: linear scale; bottom: logarithmic scale; right: m/z value legend.

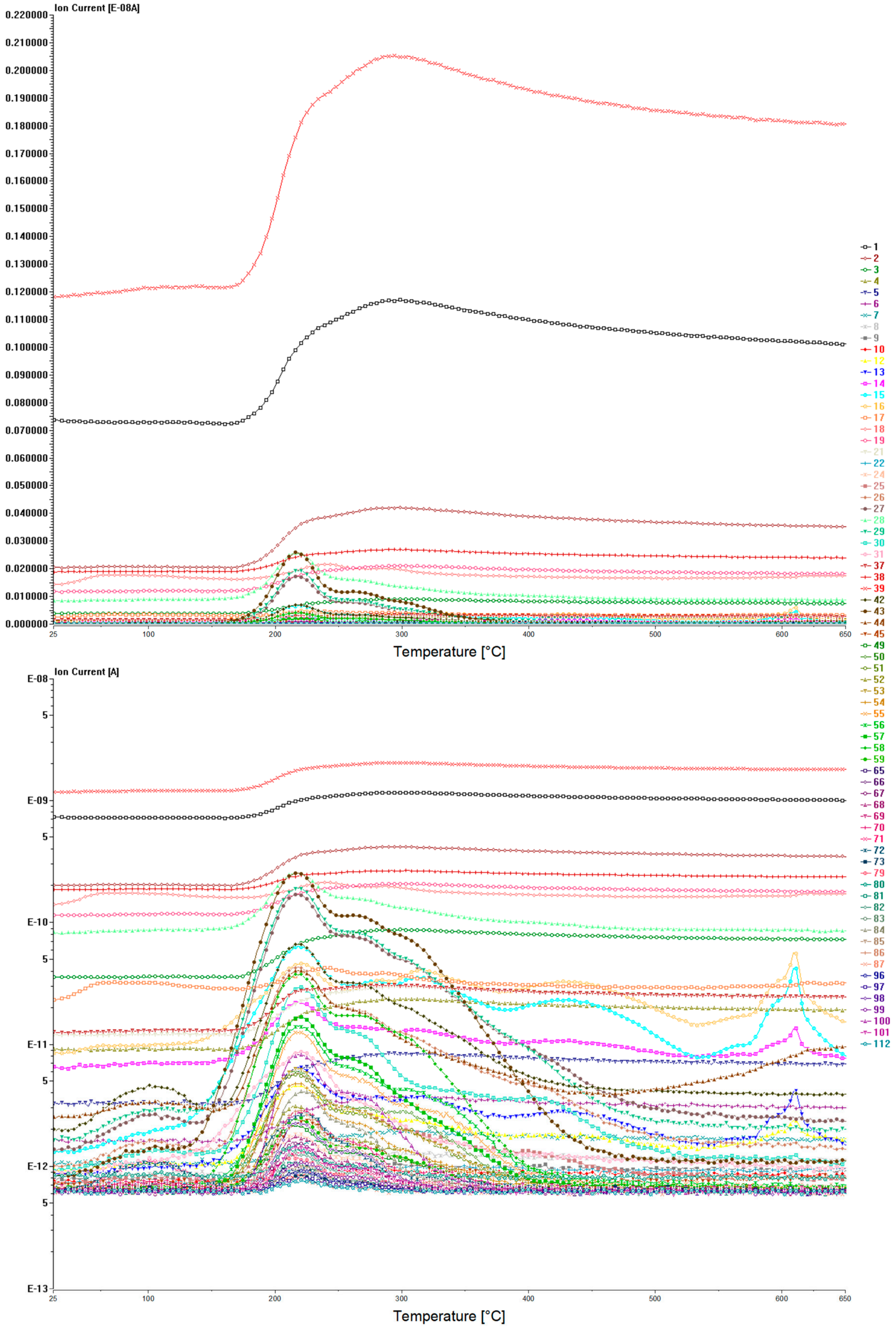


**Figure S12.** MS spectrum for **Tm** sample. Top: linear scale; bottom: logarithmic scale; right: m/z value legend.

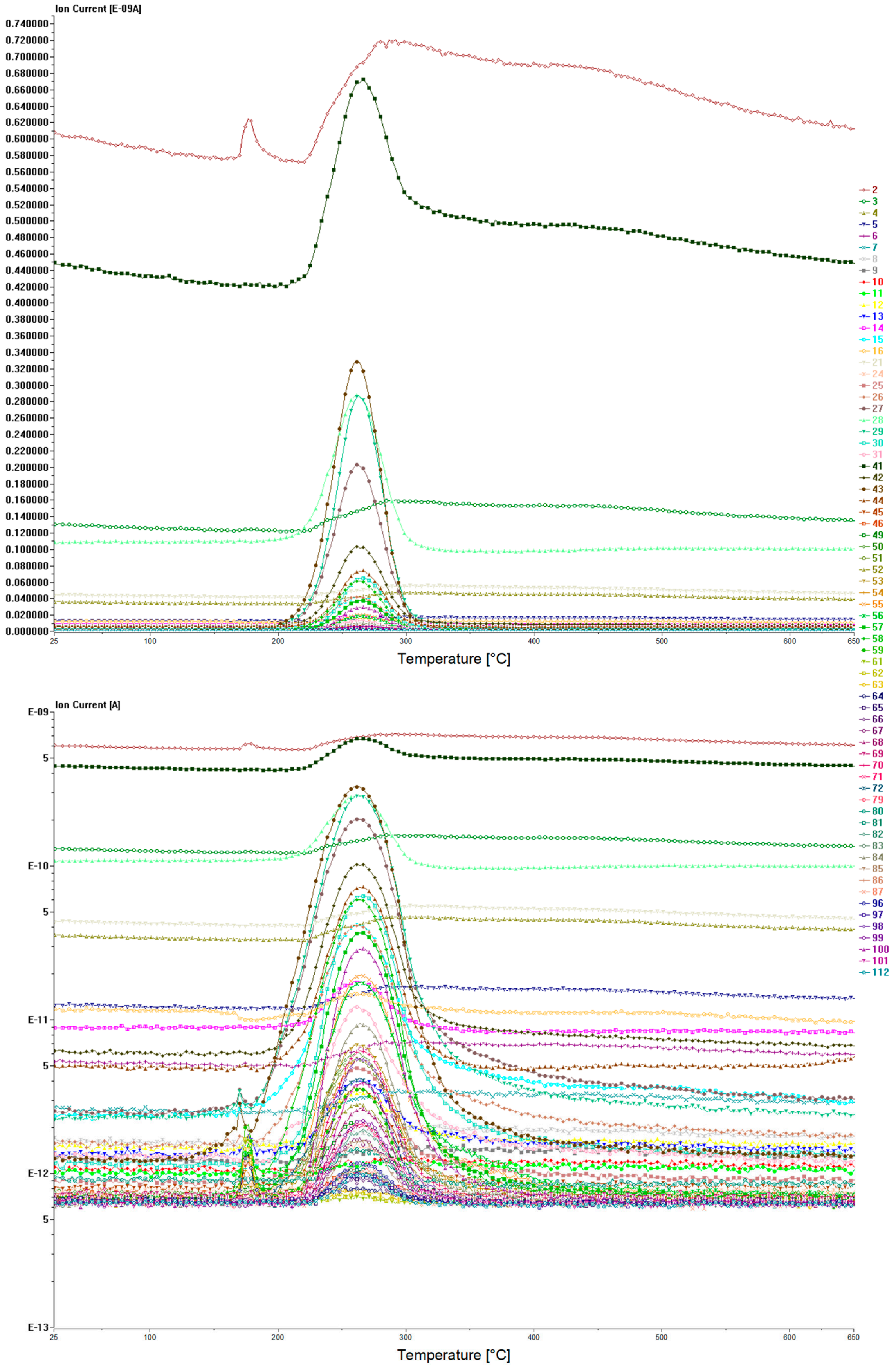


**Figure S13.** MS spectrum for **Yb** sample. Top: linear scale; bottom: logarithmic scale; right: m/z value legend.

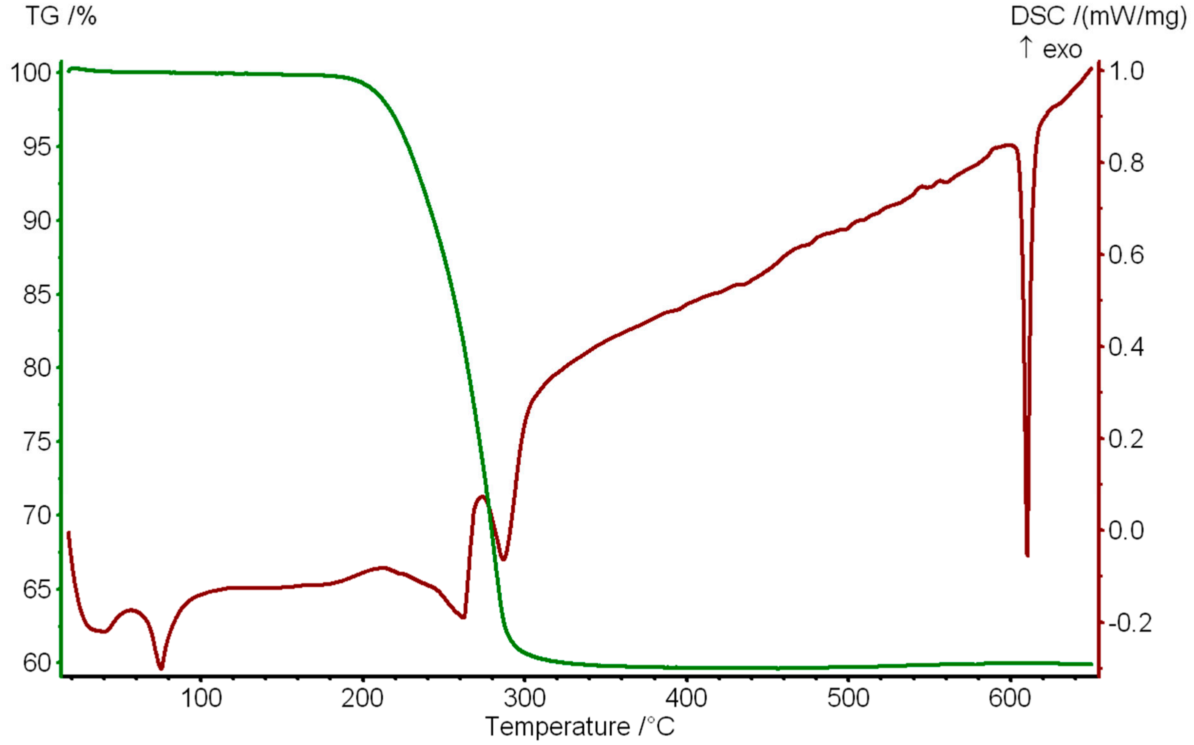


**Figure S14.** TGA/DSC curves for **Ho** sample.

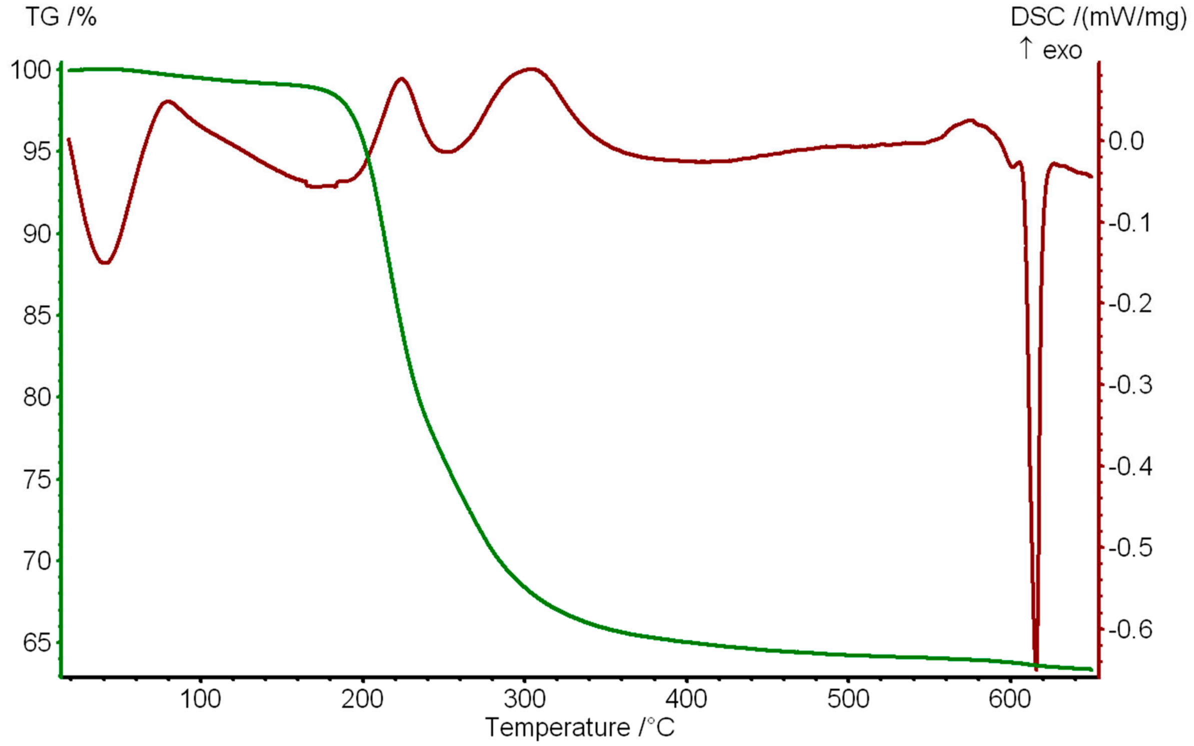


**Figure S15.** TGA/DSC curves for **Tm** sample.

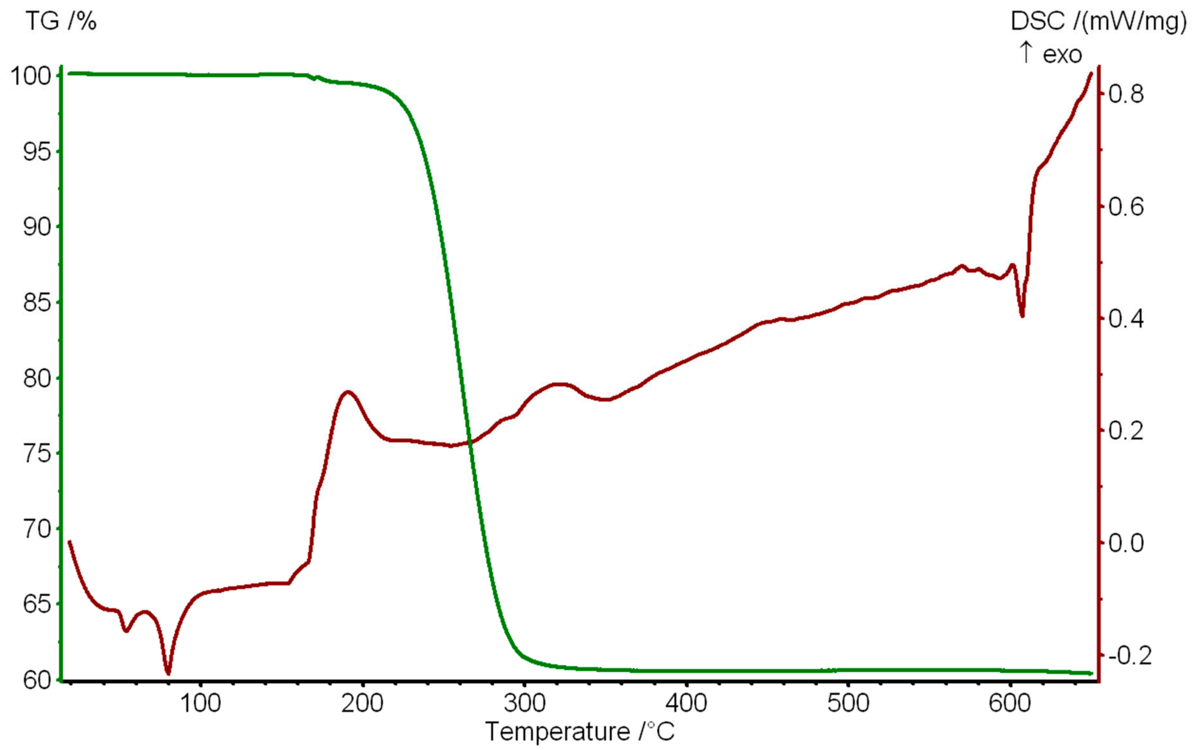


**Figure S16.** TGA/DSC curves for **Yb** sample.

## 3. Solid decomposition products

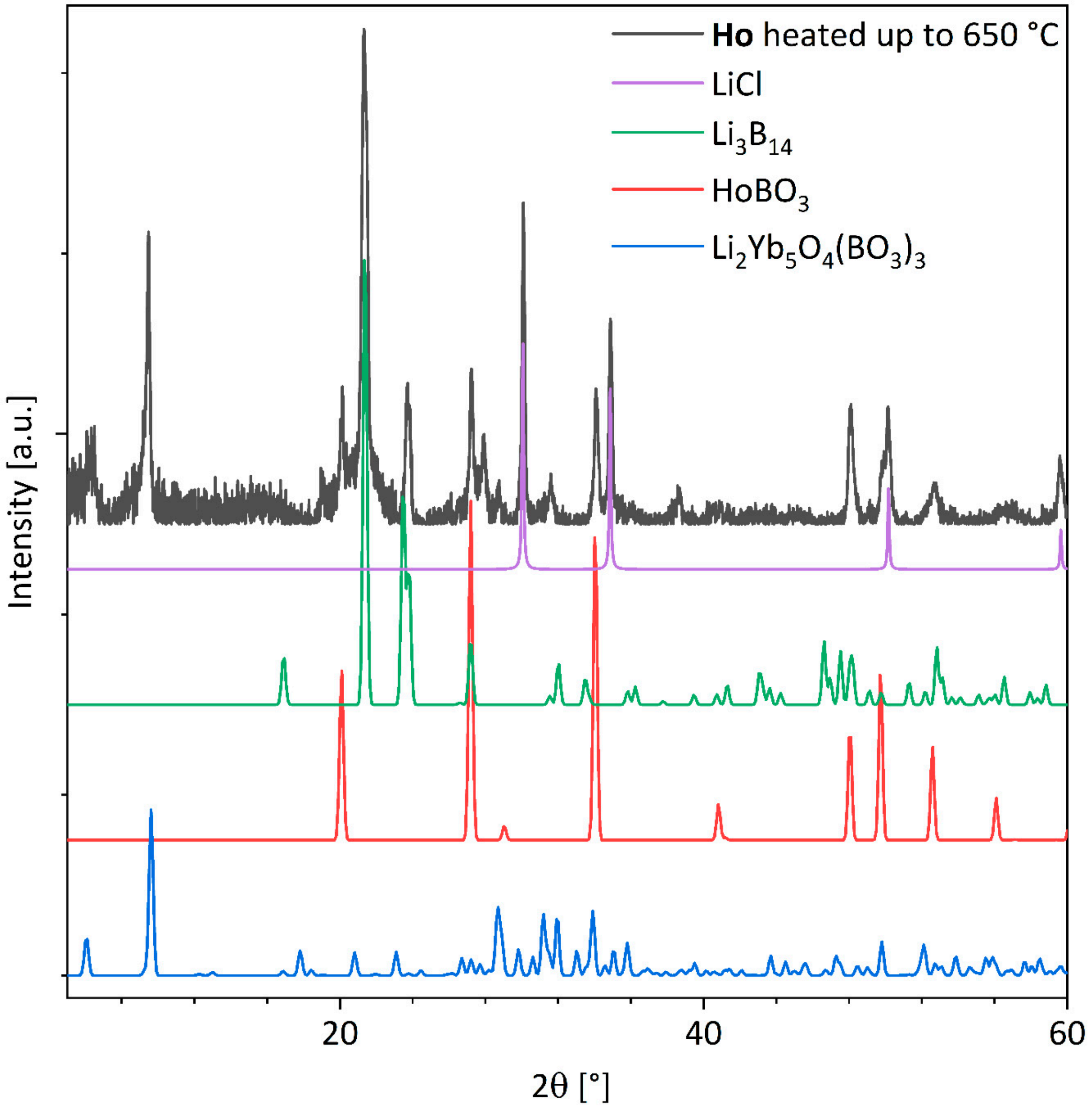


**Figure S17.** PXRD pattern (with subtracted background) of **Ho** sample heated to 650 °C, and simulated patterns of identified crystalline pyrolysis products [66-69].

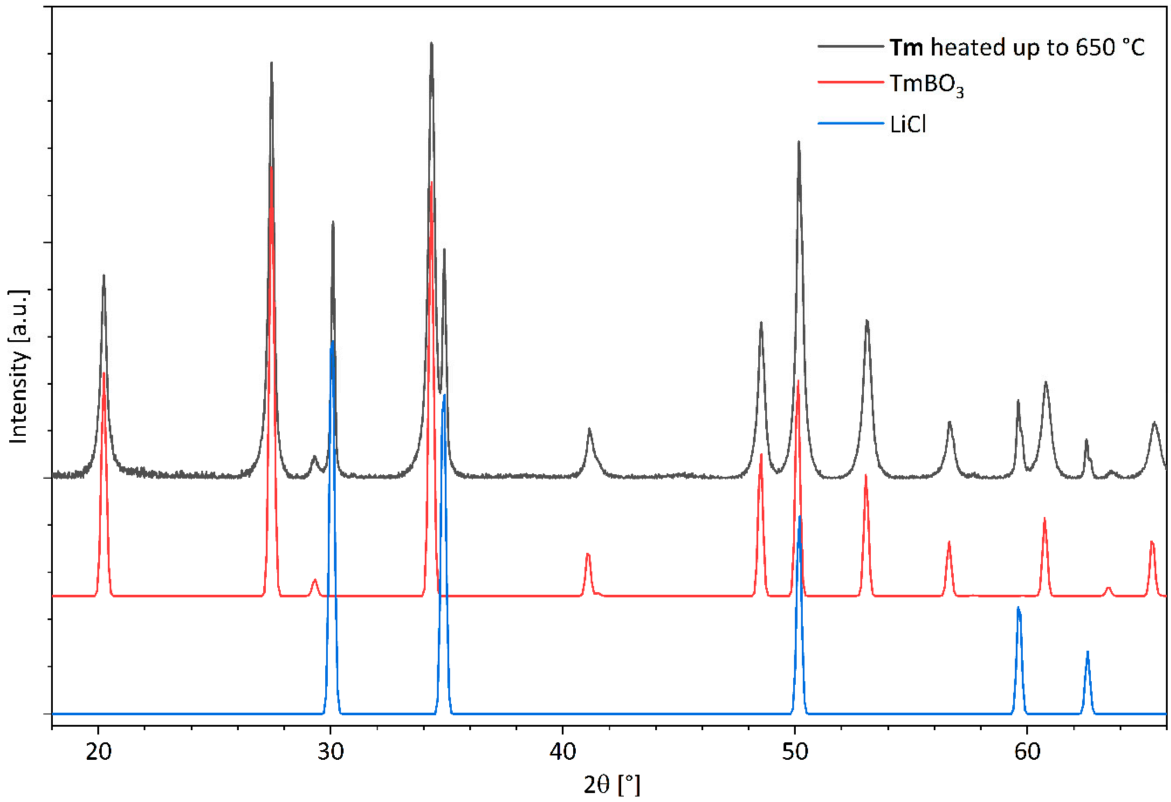


**Figure S18**. PXRD pattern (with subtracted background) of **Tm** sample heated to 650 °C, and simulated patterns of identified crystalline pyrolysis products [67,69].

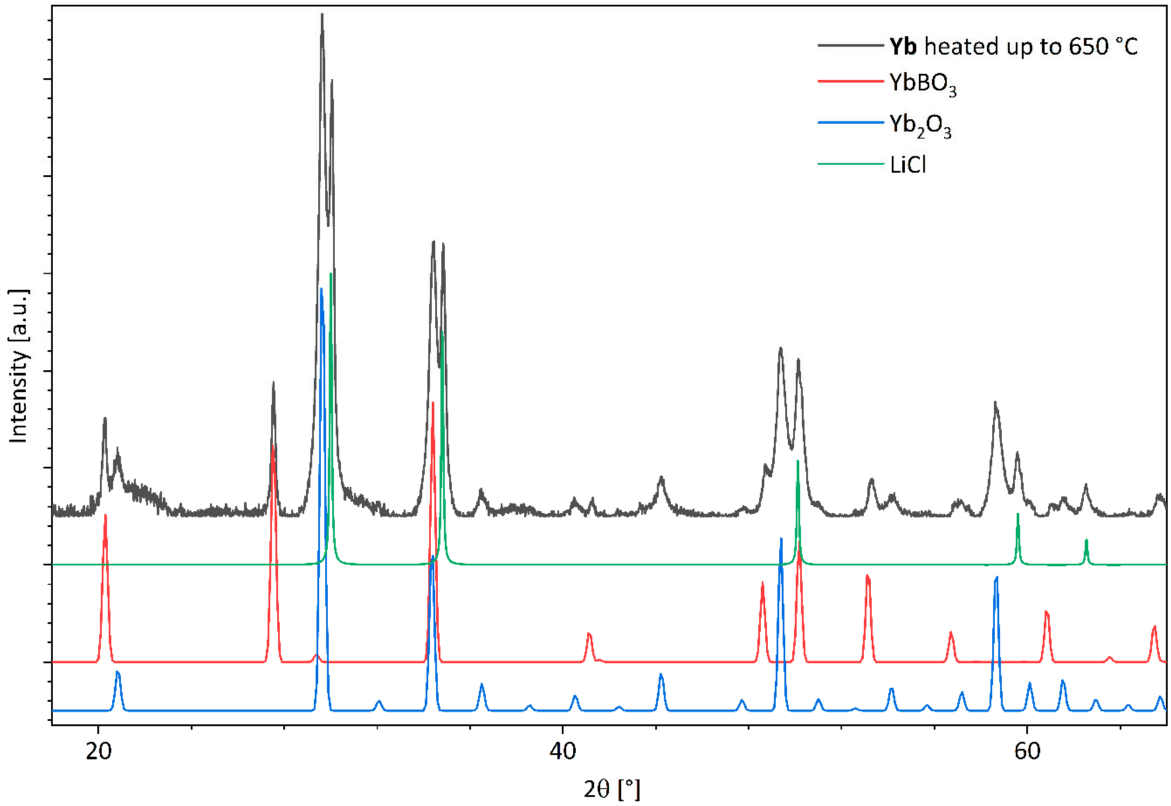


**Figure S19.** PXRD pattern (with subtracted background) of **Yb** sample heated to 650 °C, and simulated patterns of identified crystalline pyrolysis products [67,69,70].

## 4. Preliminary CIF of $\alpha$-TBAHoB from SC-XRD data (100 K)

```
data_Ho_3
_audit_creation_date              2020-12-14
_audit_creation_method
;
Olex2 1.2
(compiled 2018.05.29 svn.r3508 for OlexSys, GUI svn.r5506)
;
_audit_contact_author_address     ?
_audit_contact_author_email       ?
_audit_contact_author_name        ''
_audit_contact_author_phone       ?
_publ_contact_author_id_orcid     ?
_publ_section_references
;
Bourhis, L.J., Dolomanov, O.V., Gildea, R.J., Howard, J.A.K., Puschmann, H.
 (2015). Acta Cryst. A71, 59-75.

Dolomanov, O.V., Bourhis, L.J., Gildea, R.J, Howard, J.A.K. & Puschmann, H.
 (2009), J. Appl. Cryst. 42, 339-341.

Sheldrick, G.M. (2015). Acta Cryst. A71, 3-8.
;
_chemical_formula_moiety          'B4 H16 Ho, C16 H36 N'
_chemical_formula_sum             'C16 H52 B4 Ho N'
_chemical_formula_weight          466.83
_chemical_oxdiff_formula          'Ho B4 N C16 H52'
_chemical_oxdiff_usercomment      '100 K '
loop_
  _atom_type_symbol
  _atom_type_scat_dispersion_real
  _atom_type_scat_dispersion_imag
  _atom_type_scat_Cromer_Mann_a1
  _atom_type_scat_Cromer_Mann_a2
  _atom_type_scat_Cromer_Mann_a3
  _atom_type_scat_Cromer_Mann_a4
  _atom_type_scat_Cromer_Mann_b1
  _atom_type_scat_Cromer_Mann_b2
  _atom_type_scat_Cromer_Mann_b3
  _atom_type_scat_Cromer_Mann_b4
  _atom_type_scat_Cromer_Mann_c
```

```
   _atom_type_scat_source
   _atom_type_scat_dispersion_source
 H 0.00000 0.00000 0.49300 0.32291 0.14019 0.04081 10.51090 26.12570 3.14236
 57.79970 0.0030380000826
 'International Tables Volume C Table 6.1.1.4 (pp. 500-502)'
 'Henke, Gullikson and Davis, At. Data and Nucl. Data Tables, 1993, 54, 2'
 C 0.01920 0.00962 2.31000 1.02000 1.58860 0.86500 20.84390 10.20750 0.56870
 51.65120 0.215599998832
 'International Tables Volume C Table 6.1.1.4 (pp. 500-502)'
 'Henke, Gullikson and Davis, At. Data and Nucl. Data Tables, 1993, 54, 2'
 B 0.00963 0.00408 2.05450 1.33260 1.09790 0.70680 23.21850 1.02100 60.34980
 0.14030 -0.193200007081
 'International Tables Volume C Table 6.1.1.4 (pp. 500-502)'
 'Henke, Gullikson and Davis, At. Data and Nucl. Data Tables, 1993, 54, 2'
 Ho -15.55474 4.01359 26.90490 17.29400 14.55830 3.63837 2.07051 0.19794 11.44070
 92.65660 4.56795978546
 'International Tables Volume C Table 6.1.1.4 (pp. 500-502)'
 'Henke, Gullikson and Davis, At. Data and Nucl. Data Tables, 1993, 54, 2'
 N 0.03256 0.01839 12.21260 3.13220 2.01250 1.16630 0.00570 9.89330 28.99750
 0.58260 -11.5290002823
 'International Tables Volume C Table 6.1.1.4 (pp. 500-502)'
 'Henke, Gullikson and Davis, At. Data and Nucl. Data Tables, 1993, 54, 2'

_space_group_crystal_system         'monoclinic'
_space_group_IT_number               14
_space_group_name_H-M_alt            'P 1 21/n 1'
_space_group_name_Hall               '-P 2ybc (x-z,y,z)'
loop_
   _space_group_symop_id
   _space_group_symop_operation_xyz
 1 x,y,z
 2 -x+1/2,y+1/2,-z+1/2
 3 -x,-y,-z
 4 x-1/2,-y-1/2,z-1/2

_symmetry_Int_Tables_number          14
_cell_length_a                      11.0380(13)
_cell_length_b                      20.019(2)
_cell_length_c                      11.7510(13)
_cell_angle_alpha                   90
_cell_angle_beta                    99.643(12)
_cell_angle_gamma                    90
```

_cell_volume 2559.9(5)
_cell_formula_units_Z 4
_cell_measurement_reflns_used 2465
_cell_measurement_temperature 100.01(10)
_cell_measurement_theta_max 53.3510
_cell_measurement_theta_min 4.1310
_exptl_absorpt_coefficient_mu 5.705
_exptl_absorpt_correction_T_max 0.11351
_exptl_absorpt_correction_T_min 0.04401
_exptl_absorpt_correction_type sphere
_exptl_absorpt_process_details
;
CrysAlisPro 1.171.39.46 (Rigaku Oxford Diffraction, 2018)
Spherical absorption correction
 using equivalent radius and absorption coefficient.
Empirical absorption correction using spherical harmonics,
 implemented in SCALE3 ABSPACK scaling algorithm.
;
_exptl_crystal_colour 'clear colourless'
_exptl_crystal_colour_lustre clear
_exptl_crystal_colour_modifier .
_exptl_crystal_colour_primary colourless
_exptl_crystal_density_diffrn 1.2111
_exptl_crystal_description block
_exptl_crystal_F_000 907.1993
_diffrn_reflns_av_R_equivalents 0.1682
_diffrn_reflns_av_unetI/netI 0.1386
_diffrn_reflns_limit_h_max 11
_diffrn_reflns_limit_h_min -11
_diffrn_reflns_limit_k_max 15
_diffrn_reflns_limit_k_min -21
_diffrn_reflns_limit_l_max 12
_diffrn_reflns_limit_l_min -10
_diffrn_reflns_number 10865
_diffrn_reflns_theta_full 55.7871
_diffrn_reflns_theta_max 55.79
_diffrn_reflns_theta_min 4.41
_diffrn_ambient_environment N~2~
_diffrn_ambient_temperature 100.01(10)
_diffrn_detector 'CCD plate'
_diffrn_detector_area_resol_mean 5.2687
_diffrn_detector_type Atlas

_diffrn_measured_fraction_theta_full 0.9746

_diffrn_measured_fraction_theta_max 0.9746

_diffrn_measurement_details

;

List of Runs (angles in degrees, time in seconds):

| # | Type | Start | End | Width | t~exp~ | \w | \q | \k | \f | Frames |
|---|---|---|---|---|---|---|---|---|---|---|
| 1 | \w | -84.00 | -39.00 | 1.00 | 4.00 | -- | 0.00 | 178.00 | 150.00 | 45 |
| 2 | \w | -22.00 | 71.00 | 1.00 | 4.00 | -- | 0.00 | 38.00 | 90.00 | 93 |
| 3 | \w | -22.00 | 71.00 | 1.00 | 4.00 | -- | 0.00 | 38.00 | -180.00 | 93 |
| 4 | \w | 32.00 | 106.00 | 1.00 | 16.00 | -- | 68.24 | -99.00 | 30.00 | 74 |
| 5 | \w | -2.00 | 94.00 | 1.00 | 16.00 | -- | 68.24 | -57.00 | 90.00 | 96 |
| 6 | \w | 54.00 | 112.00 | 1.00 | 16.00 | -- | 68.24 | -125.00 | -180.00 | 58 |
| 7 | \w | -2.00 | 94.00 | 1.00 | 16.00 | -- | 68.24 | -57.00 | -180.00 | 96 |
| 8 | \w | -3.00 | 90.00 | 1.00 | 16.00 | -- | 68.24 | -38.00 | -120.00 | 93 |
| 9 | \w | -8.00 | 104.00 | 1.00 | 16.00 | -- | 68.24 | -19.00 | 120.00 | 112 |
| 10 | \w | -3.00 | 90.00 | 1.00 | 16.00 | -- | 68.24 | -38.00 | 0.00 | 93 |
| 11 | \w | 32.00 | 77.00 | 1.00 | 16.00 | -- | 68.24 | -99.00 | -30.00 | 45 |

;

_diffrn_measurement_device 'four-circle diffractometer'

_diffrn_measurement_device_type

'SuperNova, Single source at offset/far, Atlas'

_diffrn_measurement_method '\w scans'

_diffrn_orient_matrix_type

'CrysAlisPro convention (1999,Acta A55,543-557)'

_diffrn_orient_matrix_UB_11 -0.1279046000

_diffrn_orient_matrix_UB_12 -0.0124396000

_diffrn_orient_matrix_UB_13 0.0318861000

_diffrn_orient_matrix_UB_21 -0.0260076000

_diffrn_orient_matrix_UB_22 -0.0531177000

_diffrn_orient_matrix_UB_23 -0.0958200000

_diffrn_orient_matrix_UB_31 0.0546008000

_diffrn_orient_matrix_UB_32 -0.0543211000

_diffrn_orient_matrix_UB_33 0.0865516000

_diffrn_radiation_monochromator mirror

_diffrn_radiation_probe x-ray

_diffrn_radiation_type 'Cu K\a'

_diffrn_radiation_wavelength 1.54184

_diffrn_source 'micro-focus sealed X-ray tube'

_diffrn_source_type 'SuperNova (Cu) X-ray Source'

_reflns_Friedel_coverage 0.0

_reflns_limit_h_max 7
_reflns_limit_h_min -11
_reflns_limit_k_max 21
_reflns_limit_k_min 0
_reflns_limit_l_max 12
_reflns_limit_l_min -7
_reflns_number_gt 1741
_reflns_number_total 3228
_reflns_odcompleteness_completeness 98.43
_reflns_odcompleteness_iscentric 1
_reflns_odcompleteness_theta 54.18
_reflns_threshold_expression I>=2u(I)
_computing_cell_refinement 'CrysAlisPro 1.171.39.46 (Rigaku OD, 2018)'
_computing_data_collection 'CrysAlisPro 1.171.39.46 (Rigaku OD, 2018)'
_computing_data_reduction 'CrysAlisPro 1.171.39.46 (Rigaku OD, 2018)'
_computing_molecular_graphics 'Olex2 (Dolomanov et al., 2009)'
_computing_publication_material 'Olex2 (Dolomanov et al., 2009)'
_computing_structure_refinement 'olex2.refine (Bourhis et al., 2015)'
_computing_structure_solution 'ShelXT (Sheldrick, 2015)'
_refine_diff_density_max 5.7607
_refine_diff_density_min -3.1144
_refine_diff_density_rms 0.4787
_refine_ls_d_res_high 0.9322
_refine_ls_d_res_low 10.0270
_refine_ls_goodness_of_fit_ref 1.6461
_refine_ls_hydrogen_treatment mixed
_refine_ls_matrix_type full
_refine_ls_number_constraints 53
_refine_ls_number_parameters 252
_refine_ls_number_reflns 3228
_refine_ls_number_restraints 40
_refine_ls_R_factor_all 0.2576
_refine_ls_R_factor_gt 0.1996
_refine_ls_restrained_S_all 1.6352
_refine_ls_shift/su_max 0.0767
_refine_ls_shift/su_mean 0.0038
_refine_ls_structure_factor_coef Fsqd
_refine_ls_weighting_details
'w=1/[\s^2^(Fo^2^)+(0.2P)^2^] where P=(Fo^2^+2Fc^2^)/3'
_refine_ls_weighting_scheme calc
_refine_ls_wR_factor_gt 0.4724
_refine_ls_wR_factor_ref 0.5140

_olex2_refinement_description
;
1. Fixed Uiso
At 1.2 times of:
All C(H,H) groups
At 1.5 times of:
All C(H,H,H) groups
2. Restrained distances
B00L-H5aa \\sim B00L-Hl \\sim B00L-Hq \\sim B00L-Hp \\sim B00M-H6aa \\sim
B00M-Hf \\sim B00M-Hi ~
B00I-Hh \\sim B00I-H3aa \\sim B00I-Hj \\sim B00I-Hm \\sim B00M-Hn \\sim
B00J-Ho \\sim B00J-H4aa \\sim B00J-
Hg \\sim B00J-Hk
with sigma of 0.01
3. Restrained angles
Hk-B00J-Ho
fixed at 109.471 with sigma of 0.02
Hk-B00J-H4aa
fixed at 109.471 with sigma of 0.02
Hk-B00J-Hg
fixed at 109.471 with sigma of 0.02
Ho-B00J-H4aa
fixed at 109.471 with sigma of 0.02
Ho-B00J-Hg
fixed at 109.471 with sigma of 0.02
H4aa-B00J-Hg
fixed at 109.471 with sigma of 0.02
H3aa-B00I-Hj
fixed at 109.471 with sigma of 0.02
H3aa-B00I-Hm
fixed at 109.471 with sigma of 0.02
H3aa-B00I-Hh
fixed at 109.471 with sigma of 0.02
Hj-B00I-Hm
fixed at 109.471 with sigma of 0.02
Hj-B00I-Hh
fixed at 109.471 with sigma of 0.02
Hm-B00I-Hh
fixed at 109.471 with sigma of 0.02
Hp-B00L-Hq
fixed at 109.471 with sigma of 0.02
Hp-B00L-Hl

fixed at 109.471 with sigma of 0.02
Hp-B00L-H5aa
fixed at 109.471 with sigma of 0.02
Hq-B00L-Hl
fixed at 109.471 with sigma of 0.02
Hq-B00L-H5aa
fixed at 109.471 with sigma of 0.02
Hl-B00L-H5aa
fixed at 109.471 with sigma of 0.02
Hf-B00M-H6aa
fixed at 109.471 with sigma of 0.02
Hf-B00M-Hi
fixed at 109.471 with sigma of 0.02
Hf-B00M-Hn
fixed at 109.471 with sigma of 0.02
H6aa-B00M-Hi
fixed at 109.471 with sigma of 0.02
H6aa-B00M-Hn
fixed at 109.471 with sigma of 0.02
Hi-B00M-Hn
fixed at 109.471 with sigma of 0.02
4. Uiso/Uaniso restraints and constraints
Uiso(Hk) = Uiso(H4aa) = Uiso(Ho) = Uiso(Hg) = Uiso(Hp) = Uiso(Hq) = Uiso(Hl) =
Uiso(H5aa) = Uiso(H6aa) = Uiso(Hf) = Uiso(Hn) = Uiso(Hi) = Uiso(Hh) =
Uiso(H3aa) = Uiso(Hj) = Uiso(Hm)
5.a Secondary CH2 refined with riding coordinates:
C003(H00a,H00b), C004(H00c,H00d), C005(H00e,H00f), C006(H00g,H00h), C007(H00i,
H00j), C008(H00k,H00l), C00A(H00p,H00q), C00B(H00r,H00s), C00E(H00z,H),
C00F(H00,Ha), C00G(H0aa,Hb), C00H(H1aa,Hc)
5.b Idealised Me refined as rotating group:
C009(H00m,H00n,H00o), C00C(H00t,H00u,H00v), C00D(H00w,H00x,H00y), C00K(H2aa,
Hd,He)
;
_atom_sites_solution_primary dual
loop_
_atom_site_label
_atom_site_type_symbol
_atom_site_fract_x
_atom_site_fract_y
_atom_site_fract_z
_atom_site_U_iso_or_equiv
_atom_site_adp_type

_atom_site_occupancy
_atom_site_refinement_flags_posn
Ho01 Ho 0.79220(19) 0.68774(10) 0.46389(19) 0.0662(11) Uani 1.000000 .
N002 N 0.2623(18) 0.6236(11) 0.3505(17) 0.053(6) Uani 1.000000 .
C003 C 0.279(2) 0.7493(14) 0.374(3) 0.059(7) Uani 1.000000 .
H00a H 0.327(2) 0.7455(14) 0.450(3) 0.071(9) Uiso 1.000000 R
H00b H 0.335(2) 0.7504(14) 0.319(3) 0.071(9) Uiso 1.000000 R
C004 C 0.326(3) 0.6174(16) 0.248(2) 0.068(8) Uani 1.000000 .
H00c H 0.267(3) 0.6279(16) 0.180(2) 0.082(10) Uiso 1.000000 R
H00d H 0.389(3) 0.6513(16) 0.255(2) 0.082(10) Uiso 1.000000 R
C005 C 0.384(3) 0.5513(16) 0.227(3) 0.078(10) Uani 1.000000 .
H00e H 0.446(3) 0.5406(16) 0.293(3) 0.094(12) Uiso 1.000000 R
H00f H 0.322(3) 0.5166(16) 0.220(3) 0.094(12) Uiso 1.000000 R
C006 C 0.446(3) 0.5525(16) 0.115(2) 0.074(9) Uani 1.000000 .
H00g H 0.470(3) 0.5073(16) 0.100(2) 0.088(11) Uiso 1.000000 R
H00h H 0.383(3) 0.5653(16) 0.051(2) 0.088(11) Uiso 1.000000 R
C007 C 0.079(2) 0.5583(16) 0.232(2) 0.065(8) Uani 1.000000 .
H00i H 0.032(2) 0.5992(16) 0.214(2) 0.078(9) Uiso 1.000000 R
H00j H 0.124(2) 0.5492(16) 0.170(2) 0.078(9) Uiso 1.000000 R
C008 C 0.198(2) 0.6894(12) 0.3501(19) 0.045(6) Uani 1.000000 .
H00k H 0.147(2) 0.6951(12) 0.2754(19) 0.054(7) Uiso 1.000000 R
H00l H 0.144(2) 0.6878(12) 0.4074(19) 0.054(7) Uiso 1.000000 R
C009 C -0.091(3) 0.4830(15) 0.138(3) 0.080(10) Uani 1.000000 .
H00m H -0.045(4) 0.463(10) 0.085(8) 0.120(14) Uiso 1.000000 GR
H00n H -0.152(13) 0.452(9) 0.156(4) 0.120(14) Uiso 1.000000 GR
H00o H -0.131(16) 0.523(2) 0.104(11) 0.120(14) Uiso 1.000000 GR
C00A C 0.207(3) 0.8140(13) 0.365(3) 0.063(8) Uani 1.000000 .
H00p H 0.161(3) 0.8181(13) 0.287(3) 0.075(9) Uiso 1.000000 R
H00q H 0.147(3) 0.8113(13) 0.417(3) 0.075(9) Uiso 1.000000 R
C00B C 0.169(2) 0.5660(15) 0.349(2) 0.064(8) Uani 1.000000 .
H00r H 0.213(2) 0.5244(15) 0.365(2) 0.077(9) Uiso 1.000000 R
H00s H 0.121(2) 0.5733(15) 0.410(2) 0.077(9) Uiso 1.000000 R
C00C C 0.283(3) 0.8772(16) 0.394(3) 0.093(11) Uani 1.000000 .
H00t H 0.335(17) 0.884(7) 0.337(12) 0.139(17) Uiso 1.000000 GR
H00u H 0.229(3) 0.915(3) 0.39(2) 0.139(17) Uiso 1.000000 GR
H00v H 0.333(18) 0.873(5) 0.469(10) 0.139(17) Uiso 1.000000 GR
C00D C 0.552(2) 0.5963(14) 0.114(2) 0.064(8) Uani 1.000000 .
H00w H 0.558(10) 0.608(8) 0.036(3) 0.096(12) Uiso 1.000000 GR
H00x H 0.543(8) 0.636(4) 0.157(13) 0.096(12) Uiso 1.000000 GR
H00y H 0.626(3) 0.573(3) 0.149(14) 0.096(12) Uiso 1.000000 GR
C00E C 0.310(2) 0.6162(13) 0.572(2) 0.052(7) Uani 1.000000 .
H00z H 0.259(2) 0.5773(13) 0.578(2) 0.063(8) Uiso 1.000000 R

H H 0.261(2) 0.6559(13) 0.577(2) 0.063(8) Uiso 1.000000 R
C00F C -0.007(3) 0.5009(15) 0.246(3) 0.078(9) Uani 1.000000 .
H00 H 0.042(3) 0.4620(15) 0.273(3) 0.093(11) Uiso 1.000000 R
Ha H -0.056(3) 0.5125(15) 0.305(3) 0.093(11) Uiso 1.000000 R
C00G C 0.420(3) 0.6158(17) 0.669(2) 0.077(9) Uani 1.000000 .
H0aa H 0.476(3) 0.5805(17) 0.655(2) 0.092(11) Uiso 1.000000 R
Hb H 0.463(3) 0.6581(17) 0.670(2) 0.092(11) Uiso 1.000000 R
C00H C 0.358(2) 0.6153(15) 0.463(2) 0.062(8) Uani 1.000000 .
H1aa H 0.401(2) 0.5733(15) 0.459(2) 0.074(9) Uiso 1.000000 R
Hc H 0.418(2) 0.6508(15) 0.466(2) 0.074(9) Uiso 1.000000 R
B00I B 0.654(3) 0.7768(17) 0.508(3) 0.066(9) Uani 1.000000 D
B00J B 0.685(2) 0.5817(15) 0.467(3) 0.053(8) Uani 1.000000 D
C00K C 0.378(3) 0.6048(17) 0.783(2) 0.075(9) Uani 1.000000 .
H2aa H 0.349(18) 0.560(3) 0.787(8) 0.113(14) Uiso 1.000000 GR
Hd H 0.446(5) 0.612(10) 0.844(2) 0.113(14) Uiso 1.000000 GR
He H 0.313(13) 0.635(7) 0.790(8) 0.113(14) Uiso 1.000000 GR
B00L B 0.982(2) 0.6864(18) 0.615(3) 0.075(11) Uani 1.000000 D
B00M B 0.849(3) 0.701(2) 0.273(4) 0.094(14) Uani 1.000000 D
H4aa H 0.796(7) 0.614(7) 0.473(13) 0.069(19) Uiso 1.000000 D
H6aa H 0.952(8) 0.738(6) 0.315(12) 0.069(19) Uiso 1.000000 D
Hf H 0.828(13) 0.656(6) 0.356(9) 0.069(19) Uiso 1.000000 D
H3aa H 0.704(13) 0.732(5) 0.592(9) 0.069(19) Uiso 1.000000 D
Hg H 0.654(13) 0.579(7) 0.575(6) 0.069(19) Uiso 1.000000 D
Hh H 0.726(11) 0.780(8) 0.424(9) 0.069(19) Uiso 1.000000 D
Hi H 0.866(13) 0.668(7) 0.175(8) 0.069(19) Uiso 1.000000 D
Hj H 0.537(6) 0.756(7) 0.461(12) 0.069(19) Uiso 1.000000 D
Hk H 0.695(14) 0.518(3) 0.426(12) 0.069(19) Uiso 1.000000 D
H5aa H 0.966(13) 0.744(4) 0.552(10) 0.069(19) Uiso 1.000000 D
Hl H 1.010(13) 0.635(5) 0.547(9) 0.069(19) Uiso 1.000000 D
Hm H 0.649(14) 0.839(3) 0.557(11) 0.069(19) Uiso 1.000000 D
Hn H 0.749(8) 0.743(6) 0.248(13) 0.069(19) Uiso 1.000000 D
Ho H 0.596(10) 0.616(6) 0.394(10) 0.069(19) Uiso 1.000000 D
Hp H 0.875(8) 0.672(7) 0.656(12) 0.069(19) Uiso 1.000000 D
Hq H 1.076(9) 0.695(7) 0.707(9) 0.069(19) Uiso 1.000000 D

loop_
_atom_site_aniso_label
_atom_site_aniso_U_11
_atom_site_aniso_U_22
_atom_site_aniso_U_33
_atom_site_aniso_U_12
_atom_site_aniso_U_13

_atom_site_aniso_U_23
Ho01 0.0520(13) 0.0827(19) 0.0601(16) -0.0003(11) -0.0017(10) -0.0014(11)
N002 0.044(12) 0.073(16) 0.040(13) -0.004(10) 0.001(10) 0.003(10)
C003 0.045(15) 0.08(2) 0.056(18) -0.002(14) 0.028(13) -0.006(14)
C004 0.062(18) 0.10(3) 0.035(15) -0.012(16) -0.006(13) 0.006(14)
C005 0.065(19) 0.09(2) 0.06(2) 0.014(17) -0.026(16) -0.001(16)
C006 0.08(2) 0.10(2) 0.030(15) 0.003(17) -0.029(14) 0.012(14)
C007 0.058(17) 0.10(2) 0.031(14) -0.004(16) -0.001(12) 0.011(14)
C008 0.033(12) 0.070(17) 0.026(13) 0.008(12) -0.009(9) -0.012(11)
C009 0.058(18) 0.09(2) 0.09(2) -0.002(15) -0.006(17) 0.016(17)
C00A 0.069(19) 0.057(18) 0.065(19) -0.000(15) 0.019(15) -0.007(14)
C00B 0.053(16) 0.09(2) 0.049(17) -0.008(15) -0.003(13) -0.002(14)
C00C 0.10(3) 0.09(2) 0.10(3) 0.04(2) 0.01(2) 0.01(2)
C00D 0.039(14) 0.10(2) 0.056(18) 0.001(14) 0.012(13) -0.006(15)
C00E 0.031(12) 0.059(17) 0.063(17) 0.002(11) -0.004(12) 0.000(13)
C00F 0.050(17) 0.08(2) 0.09(2) 0.004(15) -0.003(16) -0.002(18)
C00G 0.063(18) 0.12(3) 0.042(17) 0.003(18) 0.009(14) -0.008(16)
C00H 0.042(15) 0.10(2) 0.045(16) 0.009(14) 0.008(12) 0.001(14)
B00I 0.08(2) 0.08(3) 0.034(18) 0.012(19) 0.007(16) -0.005(15)
B00J 0.036(15) 0.08(2) 0.050(19) 0.003(14) 0.012(13) 0.001(15)
C00K 0.061(18) 0.12(3) 0.037(16) -0.004(17) -0.000(13) 0.008(15)
B00L 0.025(14) 0.13(3) 0.05(2) 0.011(16) -0.040(14) 0.001(19)
B00M 0.036(18) 0.12(4) 0.14(4) 0.002(18) 0.04(2) 0.02(3)

## 5. FTIR spectra of as-milled samples.

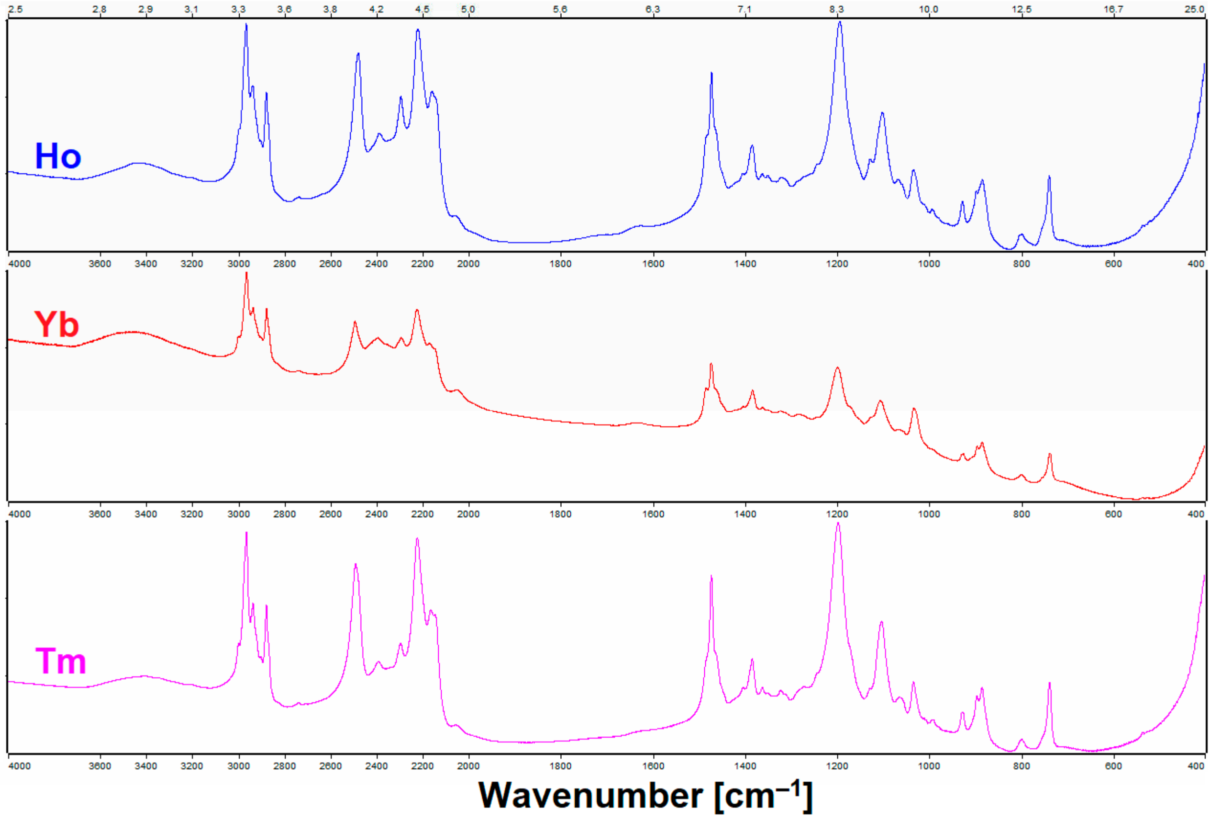


**Figure S20.** FTIR spectra of **Ho, Yb** and **Tm** as-milled samples.
